# Genomic Evolution of Influenza A(H1N1)pdm09 and A/H3N2 Viruses Among Children in Wuhan, China, Spanning the COVID-19 Pandemic (2020–2023)

**DOI:** 10.3390/v18020210

**Published:** 2026-02-05

**Authors:** Muhammad Arif Rizwan, Ying Li, Jiaming Huang, Haizhou Liu, Muhammad Noman, Ismaila Damilare Isiaka, Hebin Chen, Wenqing Li, Yuehu Liu, Huaying Wang, Yuyi Xiao, Yi Yan, Xiaoxia Lu, Di Liu

**Affiliations:** 1 State Key Laboratory of Virology and Biosafety, Wuhan Institute of Virology, Chinese Academy of Sciences, Wuhan 430071, China; marifrizwan@cuvas.edu.pk (M.A.R.); elise_lii@163.com (Y.L.); huangjm98@mail2.sysu.edu.cn (J.H.); liuhz@wh.iov.cn (H.L.); nomanuswat@mails.ucas.ac.cn (M.N.); isiakaismaila1994@gmail.com (I.D.I.); 2 University of Chinese Academy of Sciences, Beijing 101409, China; 3 Institute of Continuing Education & Extension, Cholistan University of Veterinary & Animal Sciences, Bahawalpur 63100, Pakistan; 4 Department of Respiratory Medicine, Wuhan Children’s Hospital, Tongji Medical College, Huazhong University of Science and Technology, Wuhan 430014, China; abin319@126.com (H.C.); liwenqing@zgwhfe.com (W.L.); beadoctor98@163.com (Y.L.); 18371929121@163.com (H.W.); xyy15572223229@163.com (Y.X.); 5 Pediatric Respiratory Disease Laboratory, Institute of Maternal and Child Health, Wuhan Children’s Hospital, Tongji Medical College, Huazhong University of Science and Technology, Wuhan 430014, China

**Keywords:** H1N1pdm09 and H3N2, children, COVID-19, reassortment, antigenic drift

## Abstract

Despite the persistent global threat of seasonal influenza viruses such as A(H1N1)pdm09 and A/H3N2, their epidemiological and genetic characteristics in China following the implementation of COVID-19 non-pharmaceutical interventions (NPIs) remain poorly characterized. Between September 2020 and December 2023, we conducted an integrated epidemiological and genomic analysis of influenza A viruses in children in Wuhan. The overall positivity rate for influenza A virus was markedly low at 3.43% (109/3171), reflecting a profound suppression of circulation during the pandemic. Among genotyped positives, H1N1pdm09 was predominant (52.3%), followed by H3N2 (16.5%) and untypeable strains (32.1%). Preschool children showed the highest susceptibility. Phylogenetic analysis revealed that the circulating H1N1 strains (90%) belonged to clade 6B.1A.5a.2, clustering with viruses from Hong Kong and Pakistan. In contrast, H3N2 strains (76.92%) primarily fell into clade 3C.2a1b.2a.2b, closely related to contemporary strains from Europe and North America. Notably, we identified key hemagglutinin mutations associated with antigenic drift (e.g., R240Q in H1N1; E78G, R158G in H3N2) and neuraminidase mutations potentially conferring antiviral resistance (e.g., S247N in H1N1; S245N, a putative novel glycosylation site, in H3N2). Evidence of reassortment events was also detected, underscoring the continued genomic evolution of these viruses despite their low prevalence. Our findings demonstrate that genetically diverse and antigenically drifted influenza A viruses continued to circulate and evolve in Wuhan during the COVID-19 pandemic, albeit at dramatically reduced levels. This highlights the critical need for sustained genomic surveillance and timely updates of vaccine compositions to pre-empt the resurgence of influenza in the post-pandemic era.

## 1. Introduction

Influenza viruses remain a significant cause of respiratory infections worldwide, posing a substantial threat to public health. An estimated one billion instances of seasonal influenza occur each year, with 3–5 million of those cases resulting in severe disease. Every year, it results in between 290,000 and 650,000 respiratory fatalities [1]. Children, especially those under five years old, carry a disproportionately elevated disease burden and are highly vulnerable to severe influenza complications due to their immunologically naïve status and smaller airway diameters [2,3,4]. Research indicates that influenza is a prevalent source of severe sequelae in children, encompassing neurological disorders that impact almost 11% of hospitalized pediatric cases, and is often exacerbated by polymicrobial co-infections that exacerbate disease severity [5,6,7]. Despite the acknowledged impact, critical research gaps persist in optimizing vaccination strategies for this demographic and fully understanding the role of various comorbidities [8,9].

Seasonal influenza is primarily driven by influenza A viruses, with subtypes A(H1N1)pdm09 and A/H3N2 being the most common circulating strains. The evolutionary dynamics of these viruses are characterized by two key processes: antigenic drift and antigenic shift [10]. Antigenic drift develops due to the gradual accumulation of nucleotide mutations in the major surface glycoproteins, hemagglutinin (HA) and neuraminidase (NA) proteins. Antigenic shift entails a rapid reassortment of genetic segments among various influenza viral strains during co-infection [11,12]. These variations allow viruses to evade the immune system, posing a constant threat and necessitating regular updates to influenza vaccines. Mutations in the HA gene are crucial for antigenic variation due to their influence on the virus’s capacity to adhere to host cells, thereby promoting entry and infection [13]. NA aids in viral replication and release, with changes potentially leading to resistance against antiviral medications [14]. Focusing on the genes associated with viral entry and replication, including HA and NA, corresponds with research that underscores their functions in facilitating virulence and transmission dynamics. A thorough comprehension of these genetic differences is essential for the development of effective vaccines and treatment approaches to seasonal influenza.

The emergence of the COVID-19 pandemic significantly transformed the global landscape of respiratory virus transmission. In China, the execution of non-pharmaceutical interventions (NPIs) to manage COVID-19, such as mask-wearing, social distancing, and travel restrictions, resulted in substantial decreases in influenza prevalence during the early phases of the pandemic [15,16,17]. This phenomenon was observed globally, with countries in Europe (France, the United Kingdom, Spain, Italy, Belgium, and Romania), Africa (e.g., Ghana), and Asia (e.g., China) reporting the near-disappearance of seasonal influenza peaks [18,19,20,21]. While these NPIs effectively suppressed influenza virus circulation, a critical question arose regarding the evolutionary trajectory of the viruses that managed to persist at low levels. Furthermore, the intense focus on COVID-19 management in Wuhan, the initial epicenter of the pandemic, inevitably led to a relative deficit in influenza surveillance during this period [22]. Therefore, the genetic characteristics, evolutionary dynamics, and antigenic profiles of the influenza A viruses that continued to circulate in Wuhan throughout the pandemic remain poorly elucidated, creating a significant gap in our understanding of influenza epidemiology and evolution under such unique public health conditions [22].

Consequently, to address these gaps, we conducted an integrated epidemiological and genomic study of influenza A(H1N1)pdm09 and A/H3N2 viruses in Wuhan from September 2020 to December 2023. This period encompasses the height of the COVID-19 pandemic and the subsequent lifting of restrictions, providing a unique opportunity to study influenza virus evolution under fluctuating transmission pressures. By leveraging real-time RT-PCR and whole-genome sequencing, we aimed to characterize the phylogenetic relationships, identify antigenically relevant mutations and reassortment events, and assess potential antiviral resistance in the circulating strains. Our findings provide crucial insights into the evolutionary forces shaping influenza viruses during a period of unprecedented public health intervention and are essential for informing future vaccine policy, antiviral strategies, and pandemic preparedness as global influenza activity returns to pre-pandemic patterns.

## 2. Materials and Methods

### 2.1. Specimen Collection

The study population comprised children diagnosed with influenza-like illness (ILI) who were hospitalized at Wuhan Children’s Hospital (Tongji Medical College, Huazhong University of Science and Technology) between September 2020 and December 2023. ILI is precisely defined by WHO criteria as an acute respiratory illness characterized by a recorded temperature of ≥38 °C and cough, with onset occurring within the last 10 days [23]. Nasal swabs were promptly placed in virus transport medium tubes and preserved at −80 °C until examination. A total of 3171 ILI nasal swabs were obtained.

### 2.2. Laboratory Confirmation

#### 2.2.1. Viral RNA Extraction

The BeaverBeads™ Viral DNA/RNA Kit (Beaver, Suzhou, China) was utilized for the extraction of nucleic acids from the samples. Nucleic acid was extracted using 200 µL of cleared supernatant from nasopharyngeal fluid, adhering to the manufacturer’s protocol with minor modifications, employing an automated 32-channel extractor (BEAVER Rosetta 32, TIANGEN TGuide S32, TIANGEN, Beijing, China), and eluted in 25 µL of warm RNase-free water.

#### 2.2.2. Detection Through Taqman Probe Real-Time RT-PCR

To identify influenza A, we employed the forward primer, reverse primer, and probe to amplify the matrix gene using FLUAV-M-7-F CTTCTAACCGAGGTCGAAACGTA, FLUAV-M-161-R GGTGACAGGATTGGTCTTGTCTTTA, and FLUAV-M-49-P 5′ ROX TCAGGC CCCCTCAAAGCCGAG-BHQ2 [24] by utilizing the HiScript II One Step qRT-PCR Probe Kit (Vazyme Biotech, Nanjing, China) in a real-time PCR cycler (QuantGene 9600, BIOER, Hangzhou, China) for the PCR reaction. The assay has a limit of detection (LOD) of 10 RNA copies per reaction (or 0.1 pg RNA). Reactions were conducted under the subsequent conditions: 50 °C for 15 min; 95 °C for 30 s, followed by 45 cycles of 95 °C for 10 s, and then 60 °C for 30 s.

#### 2.2.3. Single-Step RT-PCR for Multi-Amplification

Viral RNA from samples demonstrated a robust positive result for influenza A virus (IAV) (*n*: 52, with amplification achieved for *n* = 23), which served as a template for the amplification of all genes from the H3N2 and H1N1pdm09 strains. The one-step RT-PCR was conducted using the HiScript^®^ II One Step RT-PCR Kit (Dye Plus) P612 Version 22.1 (Vazyme Biotech, Nanjing, China), following the manufacturer’s guidelines for cDNA synthesis and PCR amplification. The amplification primers utilized were Uni-12/Inf1 (primer A): 5′GGGGGGAGCAAAAGCAGG-3′, Uni-12/Inf3 (primer B): 5′GGGGGGAGCGAAAGCAGG3′, and Uni-13/Inf1 (primer C): 5′CGGGTTATTAGTAGAAACAAGG-3′ [25]. Amplification reactions were conducted as a one-step mix (dye plus) 12.5 µL, enzyme mix 1.25 µL, primer-A (uni-12-Inf1) 0.4 µL, primer-B (uni-12-Inf3) 0.6 µL, primer-C (uni-13-Inf1) 1 µL and RNA 12 µL at 50 °C for 30 min; 94 °C for 3 min for reverse transcription, followed by 94 °C for 30 s, 55 °C for 30 s; then 35 cycles of 72 °C for 1.3 min, 72 °C for 7 min; and finally 4 °C for hold, for amplification. The PCR result was spotted on a 2% agarose gel and subsequently purified with the gel extraction kit (AFTSpin Universal DNA Purification Kit Version: M16B01V1.0, ABclonal, Wuhan, China).

#### 2.2.4. Library Preparation and Whole Genome Sequencing

The amplified products were purified and quantified using the QIAquick 96 PCR Purification Kit (QIAGEN, Hilden, Germany) and the QubitTM dsDNA HS Assay Kit (Thermo Fisher, Waltham, MA, USA). Subsequently, we executed a sequence of processes including tagmentation of genomic DNA, amplification of libraries, purification of libraries, normalization of libraries, and dilution of libraries to the final loading concentration, utilizing the Nextera XT DNA Library Prep kit (Illumina, San Diego, CA, USA) in accordance with the provided instructions. Ultimately, we thawed the reagent cartridge (MiSeq v2 Reagent Tray 300 cycles-PE, Illumina, San Diego, CA, USA), placed the pooled libraries into the specified reservoir of the cartridge, and operated the MiSeq sequencer in accordance with the Illumina MiSeq system handbook for sequencing.

#### 2.2.5. WGS Data Analysis

Paired-end raw sequencing reads were quality-trimmed, and adapters were removed using Trimmomatic (version 0.39) with default parameters. Contigs for specific gene segments of influenza viruses were produced utilizing the *de novo* assembly module of CLC Genomics Workbench software version 20.0.2 (CLC bio, Cambridge, MA, USA). The assembled contigs were used as query sequences for BLASTn (v2.15) analysis against the influenza virus database at the GISAID EpiFlu™ database (https://www.gisaid.org) (accessed on 8 November 2024), to identify the most closely related reference strains. Final consensus sequences for each segment were generated by mapping the quality-trimmed reads to the best-hit reference genome.

### 2.3. Clinical Data and Co-Infection Status

Clinical and demographic data, including co-infection status, were retrieved from the electronic medical records of enrolled patients. The presence of bacterial, viral (other than influenza A), or *Mycoplasma pneumoniae* co-infections was determined by the hospital’s routine clinical diagnostic procedures during the patient’s admission. These standard procedures included microbiological culture, multiplex PCR panels for respiratory viruses, and specific PCR or serological tests for *M. pneumoniae*, as clinically indicated by the treating physicians. Co-infection with SARS-CoV-2 was assessed via RT-PCR following national testing guidelines.

### 2.4. Phylogenomic Analysis & Reassortment Prediction by Constructing Phylogenetic Analysis and Principal Coordinate Analysis (PCoA)

Maximum likelihood trees for each of the eight individual gene segments were constructed using MEGA software version 7.0.26 (https://www.megasoftware.net/) (accessed on 2 December 2024). The best-fit nucleotide substitution model for each segment alignment (HKY + G, Tamura 3-parameter, or Kimura 2-parameter) was selected based on the lowest Bayesian Information Criterion (BIC) score. All models incorporated a gamma distribution of among-site rate variation (with five categories) estimated from the empirical data. Branch support was assessed with 1000 bootstrap replicates. The resulting phylogenies were visualized and annotated using FigTree version 1.4.4 (http://tree.bio.ed.ac.uk/software/figtree/) (accessed on 8 December 2024).

To provide a comprehensive evolutionary context, maximum likelihood trees were constructed using a background dataset of global H1N1 and H3N2 sequences downloaded from the GISAID EpiFlu™ database (2010–2023), ensuring inclusion of both historical strains from the region and globally representative strains from WHO-recommended vaccine clades for each season. This selection strategy was designed to accurately place our local Wuhan sequences within both the recent global circulation network and the longer-term evolutionary history of each subtype. For genome-wide phylogenetic comparison and reassortment detection, the topology of the maximum likelihood tree for each of the eight gene segments was carefully examined. Incongruence, indicating potential reassortment, was defined as a Wuhan virus strain grouping within different reference clades across segment-specific trees. This incongruence was visualized using a parallel coordinate plot, where each axis represents a gene segment, and each line traces the phylogenetic clade assignment of a single strain across all segments; divergent paths visually highlight topological discordance. Finally, the evolutionary origin of putatively reassorted segments was investigated by performing BLASTn homology searches against the GISAID database, with the closest matches annotated on the respective phylogenetic trees.

In addition, an independent PCoA analysis was conducted in another dataset that included global H1N1 MP and H3N2 MP and NS sequences from the GISAID database from 2020 to January 2026 (*n* = 3351, accessed on 1 January 2026). After performing multi-sequence alignment and modification on the potential reassorted sequences of this study with the sequences of this dataset respectively, the ape package (version 5.8-1) in R language (version 4.5.1) was used to calculate the distance matrix, and the cmdscale function was used for PCoA analysis.

### 2.5. Mutational Analysis of HA and NA (H3N2 & H1N1)

Antigenic site specificity was established by mapping viral escape mutants of H1 and H3-HA strains, utilizing A/H1N1/Victoria/4897/2022 and A/H3N2/Hong Kong/4801/2014 as references, respectively. H1 and H3 numbering was executed via Librator-Win10 (http://Wilsonlab.uchicago.edu, accessed on 1 November 2025) [26]. The examination of substitutions at HA was conducted utilizing A/H1N1/Victoria/4897/2022 and A/H3N2/Hong Kong/4801/2014 as reference sequences for H1N1 and H3N2, respectively, to identify alterations at the antigenic sites and adjacent positions for the clinical samples sequenced from the year 2023. The same was done for the Neuraminidase (NA) gene. Substitutions were identified through a manual search of aligned sequences using MEGA 6.0.

### 2.6. 3-D Model Visualization of the HA Protein

The three-dimensional structures of HA proteins were predicted to visualize the HA molecules of A/H1N1/Victoria/4897/2022 and A/H3N2/Hong Kong/4801/2014 using PyMOL software 4.6.0 (https://www.pymol.org/) (accessed on 1 January 2025). The identified mutations within antigenic sites and other significant locations were marked accordingly.

### 2.7. Prediction of N-Glycosylation Sites

N-linked glycosylation sites were predicted utilizing the NetNGlyc 2.0 web server (http://www.cbs.dtu.dk/services/NetNGlyc) (accessed on 5 February 2025) with a threshold value exceeding 0.5. This server recognizes the amino acid alignment N-X-S/T, with X representing any amino acid except Aspartic acid or Proline.

### 2.8. Ethical Consideration

The institutional review board and the Ethics Committee of Wuhan Children’s Hospital approved this study (No. 2025R086-E01).

## 3. Results

### 3.1. Suppressed Influenza Activity and Shifted Epidemiology in Wuhan in the COVID-19 Era

**Figure 1:**
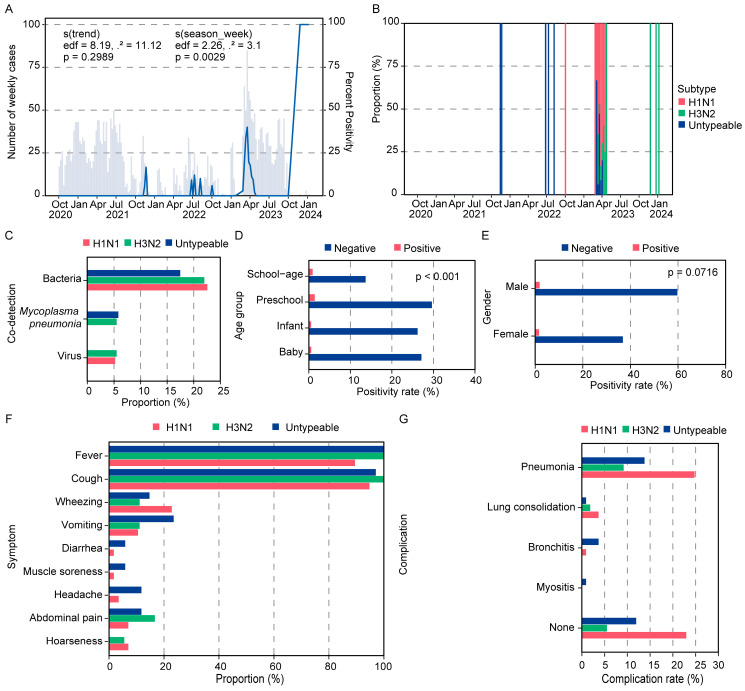
Clinical and epidemiological characteristics of influenza A virus infections among children in Wuhan, 2020–2023. (**A**) Temporal trends of Influenza A virus (IAV) detection among pediatric ILI hospitalizations. The shaded area (or bars) represents the number of weekly confirmed IAV cases (left y-axis). The solid blue line represents the weekly IAV test positivity rate. (**B**) The stacked bar chart shows the monthly proportion of IAV-positive specimens characterized as H1N1pdm09 (pink), H3N2 (green), or untypeable (blue, indicating IAV-positive but subtyping not determined) from October 2020 through January 2024. (**C**) Proportions of co-detected pathogens across subtypes. (**D**) Age-group–specific positivity rates among pediatric patients. (**E**) Sex distribution of IAV positivity. (**F**) Frequency of clinical symptoms across subtypes. (**G**) Distribution of complications associated with IAV infection. The generalized additive model (GAM) was applied to assess seasonal trends in IAV detection.

Out of a total of 3171 samples analyzed, 109 tested positive for Influenza A virus (IAV), with the vast majority (96.56%) yielding negative results, indicative of substantially suppressed influenza activity during the study period (Table 1). Temporal analysis using a generalized additive model (GAM) revealed a discernible disruption seasonal pattern in IAV detection, marked by specific weekly variations, with no notable long-term upward or downward trend observed during the research period (Figure 1A). Temporal analysis revealed a significant resurgence of IAV activity in the final months of the surveillance period (October 2023–January 2024), marked by a sharp increase in test positivity rates (Figure 1A). This pattern suggests intense virus circulation during this phase, with the divergence between rising positivity and case counts potentially indicative of a transition to more focused diagnostic testing in clinical settings. Among the identified viral genotypes, H1N1 was the most predominant, comprising 52.3% of cases, followed by untypeable cases at 31.2% (genotyping attempt unsuccessful or sample quality inadequate for genotyping) and H3N2 at 16.5% (Figure 1B). ‘Untypeable’ indicated they were positive for influenza A matrix gene but could not be subtyped by multiplex RT-PCR or sequenced, likely due to low viral load from late presentation or suboptimal sample quality. Their clinical profiles are presented in Table 1, but should be interpreted with this caveat. This distribution may indicate fundamental disparities in transmission dynamics, pathogenicity, or geographic frequency among the viral types and underscore the necessity for additional exploration of the biological or environmental factors affecting these patterns. Analysis of clinical records for coinfection revealed that bacterial coinfection was the predominant form across all viral categories, with prevalence rates ranging from 17.6% to 22.8%, and was most frequent in H1N1 cases. Viral coinfections were infrequent (5.3–5.6%) and conspicuously missing in untyped cases. Mycoplasma pneumoniae was identified solely in conjunction with H3N2 and untyped cases (5.6–5.9%). Testing for SARS-CoV-2 was performed for all ILI admissions. No cases of influenza A and SARS-CoV-2 co-infection (0/109) were identified among the study groups. (Figure 1C).

Stratification by age group revealed that preschool children (3–6 years old) exhibited the highest testing volume (31.03%) and the highest positive rate (1.42%), followed by school-age children (>6 years old, 0.91%). Infants and babies (<1 and 1–3 years old, respectively) were evaluated at comparable rates (~27% each) but showed lower positive rates (0.57% and 0.54%, respectively) (Figure 1D). This pattern indicates age-related susceptibility, with preschool-aged children potentially at higher risk of infection. Despite these variations, the overall positivity rate across all pediatric cohorts remained low (<1.5%), reflecting a minimal disease burden in this population during the study. A gender imbalance was observed in testing, with males accounting for 61.58% of samples compared to 38.42% for females. Males also showed a marginally higher positivity rate (1.84% vs. 1.62%) (Figure 1E).

Symptom profiling of confirmed cases (*n* = 109) indicated unique clinical presentations among the three viruses’ subtypes (Table 1). Fever and cough were almost universally seen symptoms (present in 89–100% of cases), confirming their significance as primary indications of infection. Untypeable cases exhibited greater systemic involvement, with markedly elevated incidences of vomiting (23.5%), headache (11.8%), and abdominal pain (11.8%) relative to other viral types (*p* < 0.05). H3N2 demonstrated a distinctive prevalence of stomach pain (16.7%). Wheezing was predominantly linked to H1N1 (22.8%), whilst hoarseness was mainly noted in instances of H1N1 and H3N2. Less prevalent symptoms, including diarrhea and muscle discomfort (<6%), were mentioned more frequently in untypeable cases (Figure 1F). These data indicate that untypeable cases are likely to cause more broad sickness, while H1N1 and H3N2 predominantly result in localized respiratory symptoms. Regarding complications, pneumonia was the most prevalent consequence among all viral types, with the highest prevalence in H1N1 cases (24.8%), followed by untyped cases (13.8%) and H3N2 (9.2%). Untyped cases had a greater incidence of bronchitis (3.7%) relative to H1N1 (0.9%), and were the sole variant associated with myositis (0.9%). A significant percentage of H1N1 infections (22.9%) resolved without any documented complications, indicating a diverse clinical spectrum that includes mild manifestations. Severe pneumonia with consolidation was noted across all viral types, occurring in 4/57 (7.01%) of H1N1, 2/18 (11.11%) of H3N2, and 1/34 (2.94%) of untypeable cases (Table 1, Figure 1G). This indicates that H1N1 was a major driver of pneumonia, untypeable cases present a wider array of sequelae, and H3N2 shows moderate clinical severity.

### 3.2. Phylogenetic Analysis Reveals Global Clade Circulation

Collectively, epidemiologic results depict a period of markedly low influenza A virus prevalence in Wuhan, consistent with the broader context of the COVID-19 pandemic. From the 109 positive samples, 23 viral isolates (10 H1N1 and 13 H3N2) were successfully amplified and subjected to whole-genome sequencing. All consensus genomes were successfully sequenced with coverages exceeding 95%, and a mean sequencing depth of 1000× to 3000× was achieved across all segments, despite a general inverse correlation between segment length and coverage (Supplementary Tables S1 and S2). These isolates serve as the basis for subsequent molecular genetic analyses.

It is worth noting that all influenza A isolates that yielded whole-genome sequences (*n* = 23) were obtained in 2023, following the relaxation of the strictest Non-Pharmaceutical Interventions (NPIs) in December 2022. The complete absence of sequenced viruses from the period of stringent lockdowns (2020–2022) is consistent with the near-suppression of community influenza transmission during that time. And the following genomic analyses, based on the 23 successfully sequenced isolates from 2023, provide a detailed snapshot of the specific variants circulating in Wuhan’s pediatric population during the late phase of the study. Their evolutionary context is inferred from comparison with the global sequence dataset (GISAID, 2010–2023).

**Figure 2:**
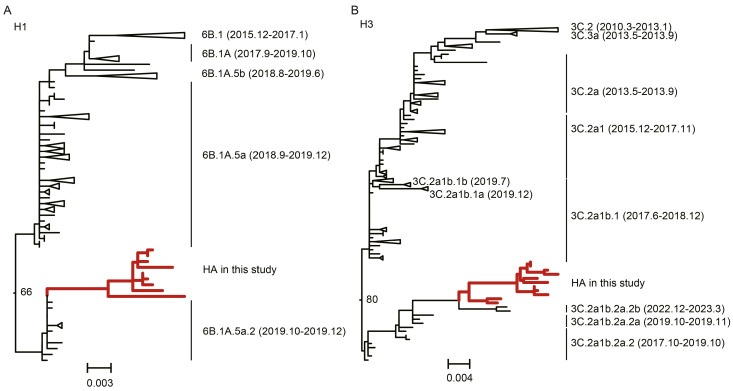
Phylogenetic analysis of influenza A/H1N1 and A/H3N2 viruses circulating in Wuhan, 2020–2023. (**A**) Maximum-likelihood phylogeny of the hemagglutinin (HA) gene of A/H1N1 viruses from Wuhan and reference strains representing major clades. (**B**) Maximum-likelihood phylogeny of the HA gene of A/H3N2 viruses from Wuhan and representative global lineages. Topologies were inferred using MEGA v7.0.26 under the maximum-likelihood method with 1000 bootstrap replicates; only nodes with bootstrap support > 70% are shown, and scale bars indicate nucleotide substitutions per site.

To elucidate the genetic origins and evolutionary relationships of the circulating strains, we performed phylogenetic analysis on the sequenced isolates. For H1N1, the majority (90%, 9/10) of the HA genes from 2023 clustered within subclade 6B.1A.5a.2, forming a close phylogenetic relationship with strains previously circulating in Hong Kong and Pakistan since 2019 (Figure 2A). A congruent topology was observed for the NA gene and most other genomic segments (PB2, PB1, PA, NP, NS), confirming their common evolutionary trajectory within clade 6B (Supplementary Figures S1–S8).

Similarly, the H3N2 collected between 2020 and 2023 were all classified within clade 3C.2a. The majority (76.92%, 10/13) of the HA genes from 2023 fell into subclade 3C.2a1b.2a.2b, demonstrating the closest genetic similarity to contemporary strains from France, Spain, and Canada (2022–2023) (Figure 2B). The phylogenetic structure of the NA gene and other inner genes (PB2, PB1, PA, NP) was largely consistent with that of the HA gene (Supplementary Figures S9–S16). The presence of minor subclades (e.g., 3C.2a1b.2a.2a.1b) suggests multiple, independent introduction events or unsampled diversity within the local transmission network.

Notably, the analysis revealed a distinct temporal origin for the two subtypes. The H1N1 viruses from Wuhan in 2023 clustered phylogenetically with global sequences from the 2019–2020 pre-pandemic period, suggesting the persistence or re-emergence of a pre-COVID lineage. Conversely, the H3N2 viruses were most closely related to strains circulating globally in 2022–2023, indicating a more recent, post-pandemic reintroduction into the region.

### 3.3. Reassortment Events Enhance Genomic Diversity

**Figure 3:**
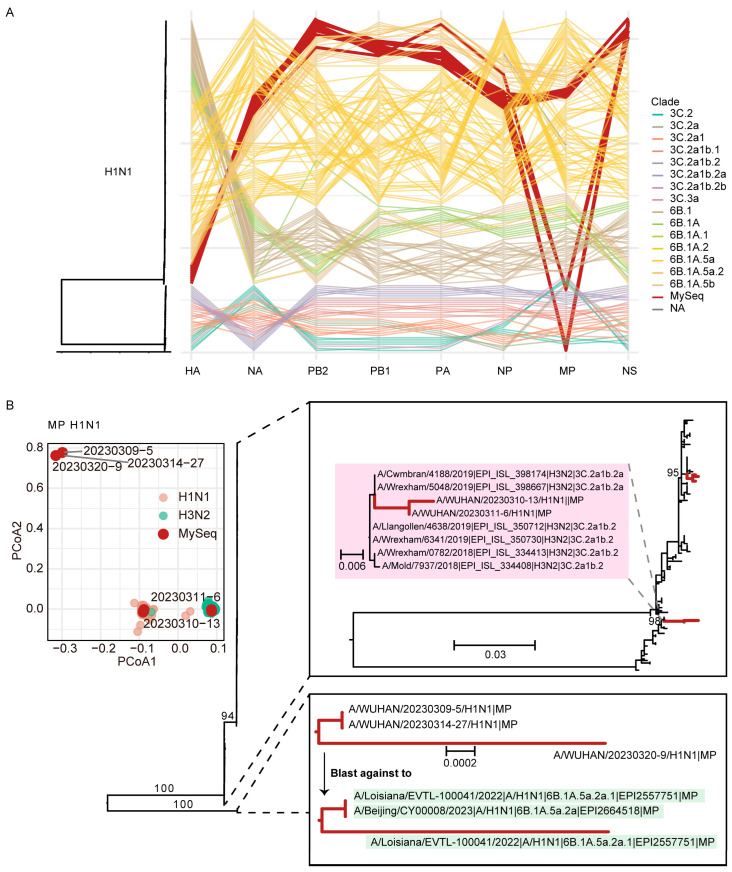
Potential reassortment events of influenza A/H1N1 viruses. (**A**) Genome-wide phylogenetic comparison of eight genomic segments of A/H1N1 isolates from Wuhan and worldwide sequences. (**B**) The phylogenetic tree represents the putative source clade of reassortment of the matrix protein (MP). The PCoA plot shows potential MP fragment reassortment events worldwide.

We investigated potential reassortment events by comparing the phylogenetic topologies of all eight genomic segments. For H1N1, incongruence was detected in the matrix protein (MP) gene (Figure 3A). The other seven segments clustered as expected with clade 6B.1A.5a. The potential reassorted MP gene of several strains showed a closer phylogenetic affinity to H3N2 of subclade 3C.2a1b.2a (e.g., a 2019 United Kingdom strain), indicating a historical reassortment event (Figure 3B).

**Figure 4:**
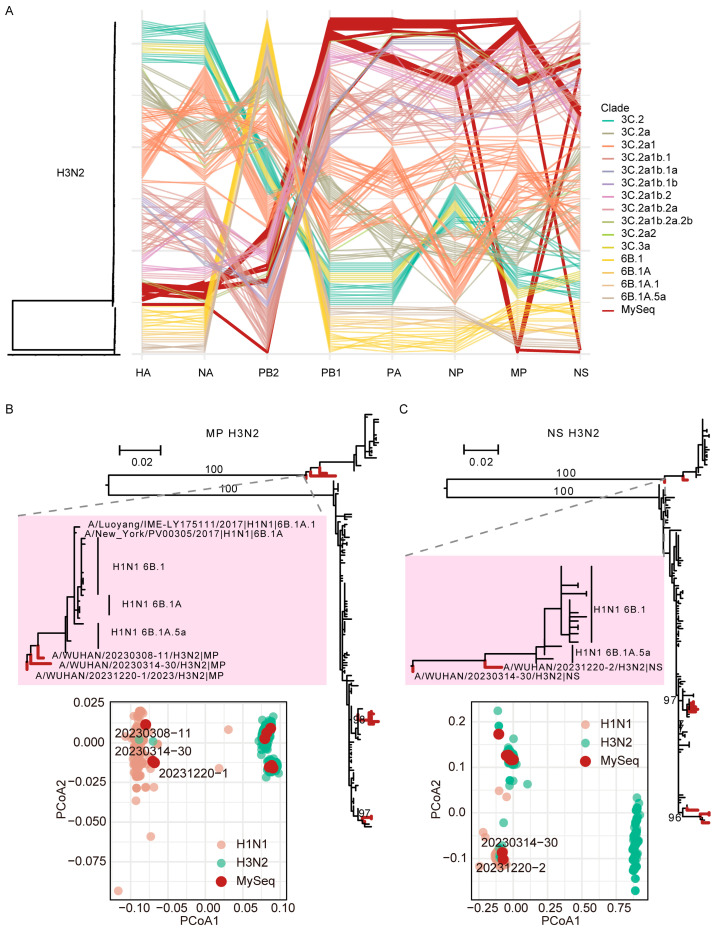
Potential reassortment events of influenza A/H3N2 viruses. (**A**) Genome-wide phylogenetic comparison of eight genomic segments of A/H3N2 isolates from Wuhan and worldwide sequences. (**B**) The phylogenetic tree represents the putative source clade of reassortment of the matrix protein (MP). The PCoA plot shows potential MP fragment reassortment events worldwide. (**C**) The phylogenetic tree represents the putative source clade of reassortment of the matrix protein (MP). The PCoA plot shows potential MP fragment reassortment events worldwide.

Reassortment was more frequent in H3N2 viruses. Incongruent phylogenies were identified for both the MP and non-structural (NS) genes (Figure 4A). Specifically, these segments from some Wuhan H3N2 strains clustered within the H1N1 clade 6B.1A.5a, closely related to strains from Bangladesh (2019), rather than with their own HA clade (Figure 4B,C). These findings underscore the role of reassortment in generating genomic diversity among co-circulating influenza viruses.

The PCoA results revealed clear, biologically interpretable reassortment patterns that are fully concordant with the maximum likelihood phylogenetic analyses (Figure 3B and Figure 4B,C). Notably, similar segment-specific reassortment patterns were also observed among globally sourced reference sequences, indicating that the detected reassortment events are not unique to our newly generated data, but rather reflect broader reassortment dynamics present in the global influenza virus population.

### 3.4. Potential Antigenic Drift Related Key Mutations in Hemagglutinin

**Figure 5:**
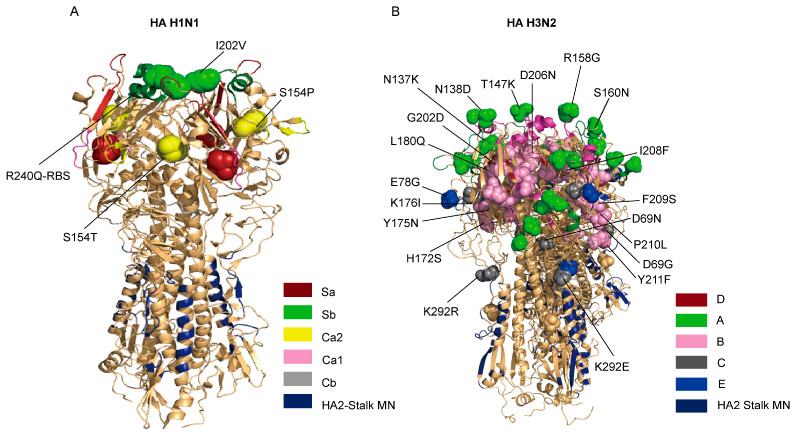
Three-dimensional (3D) structure and key amino acid substitutions in the hemagglutinin (HA) protein of influenza A viruses collected in Wuhan, 2023. (**A**) Three-dimensional (3D) structure of the HA protein of A/H1N1 viruses, showing the positions of identified amino acid substitutions. (**B**) Three-dimensional (3D) structure of the HA protein of A/H3N2 viruses, highlighting mutations located within or near known antigenic sites.

We next characterized amino acid substitutions in the HA protein that could confer antigenic drift. Compared to the A/Victoria/4897/2022 vaccine strain, the H1N1 isolates of this study from 2023 exhibited 16 amino acid changes (98.6–99.5% identity). Several of these were located in or near critical antigenic sites, including S154P/T (Ca2), I202V (Sb), and notably, R240Q, which is situated adjacent to the receptor-binding site (RBS) (Supplementary Table S3, Figure 5A). These mutations are likely to influence the antigenic properties of the virus.

The H3N2 HA protein showed more substantial divergence, with 46 amino acid substitutions (94.5–96.6% identity) relative to the A/Hong Kong/4801/2014 vaccine strain. Multiple mutations were concentrated in known antigenic epitopes, including N137K, N138D, T147K, R158G, and S160N in epitope A; I156K a cluster of changes between positions 172–211 in epitope B; and E78G in epitope E (Supplementary Table S4, Figure 5B). This accumulation of changes in key antigenic regions highlights the significant antigenic evolution of the A/H3N2 subtype.

### 3.5. Neuraminidase Mutations and Potential Antiviral Resistance

Mutations in the NA gene associated with reduced inhibitor sensitivity or altered function were identified in both subtypes. In H1N1, these included S247N, located near the enzyme’s active site and previously linked to reduced sensitivity to oseltamivir, and E382G, a putative permissive mutation for resistance (Supplementary Table S5). Oseltamivir is the first-line antiviral therapy for influenza in China [27,28,29], making the surveillance of these polymorphisms critical for informing treatment guidelines and ensuring therapeutic efficacy.

In H3N2, we observed the S245N/S247T double mutation, which has been demonstrated to diminish antibody binding and become predominant in recent global strains. Other notable mutations included R150H and V215L (near the active site), and N329S/G alongside S331G, which disrupt a conserved glycosylation site (NDS) and may affect enzymatic activity and antigenicity (Supplementary Table S6). The presence of these mutations warrants continuous monitoring of antiviral drug efficacy.

### 3.6. Shifts in Glycosylation Patterns

Analysis of potential N-linked glycosylation (NLG) sites revealed clade-specific adaptations. In H1N1 HA, a single potential site was conserved at position 293, with an adjacent substitution (NAT→NTT) that may fine-tune glycan structure. In H1N1 NA, a DKS→NSK substitution at positions 50–52 introduced a novel potential glycosylation site (Supplementary Table S7).

More dynamic changes were observed in H3N2. In the HA protein, NLG sites were lost at positions 109 and 138 due to amino acid substitutions, but a new site was acquired at position 160. In the NA protein, new NLG sites were introduced at positions 245 and 463, while the site at position 329 was lost (Supplementary Table S8). These gains and losses of surface glycans are likely to impact viral antigenicity and immune evasion by altering antibody accessibility.

## 4. Discussion

The present research provides a local genomic analysis of influenza H1N1 and H3N2 viruses that persisted in children in Wuhan during a period of unprecedented low incidence from 2020 to 2023. While our sample size is limited and geographically focused, the detection of reassortment and specific mutations within these locally circulating strains provides evidence that evolutionary processes continued even under constrained transmission conditions. These local findings highlight the need for sustained surveillance to understand how such changes integrate into broader global trends.

Despite a dramatically suppressed circulation rate of 3.43%, our data reveal that the co-circulating A(H1N1)pdm09 and A/H3N2 viruses underwent continuous evolution, evidenced by clade-specific diversification, reassortment, and the accumulation of antigenically relevant mutations. This underscores the remarkable adaptive capacity of influenza viruses even under constrained transmission conditions.

The drastically reduced influenza positivity rate observed in our study aligns with global reports of receding influenza activity due to NPIs implemented against COVID-19 [30]. Initially, hospitalized patients underwent screening for respiratory virus infections, but the complete absence of influenza B virus detection throughout the 2020–2023 surveillance period is a notable finding. This may reflect a genuinely lower community prevalence of influenza B during these seasons, a potential differential impact of NPIs on the transmission dynamics of influenza A versus B viruses, or stochastic variation due to the overall low incidence of influenza activity. The predominance of H1N1 over H3N2 during this period may reflect intrinsic differences in viral fitness or environmental stability under these specific intervention pressures. Furthermore, the distinct clinical presentation of untypeable strains, characterized by heightened systemic symptoms, warrants further investigation, as it could indicate infections with viral quasispecies or novel variants that escaped conventional genotyping, consistent with previous investigations [31].

Phylogenetic analysis confirmed that the limited viral diversity in Wuhan was not isolated but was integrally linked to global circulation networks. The predominance of clades 6B.1A.5a.2 (H1N1) and 3C.2a1b.2a.2b (H3N2) mirrors their contemporaneous dominance in Asia, Europe, and North America, suggesting ongoing international seeding events, a phenomenon supported by multiple studies [32] and prior research examining the mechanisms of viral transmission [33]. Our phylogenetic analysis revealed a notable difference in the temporal origins of the co-circulating subtypes. The H1N1 viruses appeared to descend from a pre-pandemic (2019–2020) lineage, potentially indicating cryptic local persistence or a regional reservoir that survived the period of intense NPIs. In stark contrast, the H3N2 viruses were phylogenetically linked to very recent (2022–2023) global strains, suggesting they were reintroduced following the relaxation of travel and social restrictions. This divergence underscores how different influenza A subtypes can experience distinct epidemiological trajectories under the same broad public health pressures, with H1N1 exhibiting a pattern of local continuity and H3N2 one of global migration and replacement.

Crucially, we detected potential reassortment events by comparing the phylogenetic topologies of all eight genomic segments, as visualized in genome-wide parallel coordinate plots (Figure 3A and Figure 4A). In H1N1, incongruence was detected in the matrix protein (MP) gene, which clustered with H3N2 viruses of subclade 3C.2a1b.2a, indicating historical reassortment (Figure 3A,B). In H3N2, incongruence affected both MP and non-structural (NS) genes, which grouped within the H1N1 clade 6B.1A.5a (Figure 4A–C). BLASTn analysis confirmed the inter-subtype origin of these segments. These patterns are unlikely to stem from PCR contamination, as the reassorted segments consistently grouped with geographically and temporally distinct reference strains rather than co-processed samples, and stringent negative controls remained negative throughout. The combined evidence from segment-resolved PCoA and phylogenetic analyses strongly argues against this possibility. Technical artifacts would be expected to produce inconsistent or random clustering across multiple segments; in contrast, the reassortant samples identified here exhibit highly specific, segment-limited discordance, while non-reassorted samples show highly coherent clustering across all eight segments, supporting the integrity of the dataset. In addition, while influenza virus reassortment is well documented globally, our analyses indicate that within this globally representative dataset, reassortment events are relatively infrequent and confined to specific segments and samples, rather than widespread—an observation that is biologically plausible and consistent with previous reports showing context-dependent reassortment frequencies. These findings underscore the role of reassortment in generating genomic diversity among co-circulating influenza viruses.

The antigenic drift observed in both subtypes, driven by mutations in key HA epitopes (e.g., H1N1-R240Q; H3N2-R158G, E78G), likely contributed to the viruses’ ability to evade pre-existing population immunity. This is compounded by clade-specific alterations in glycosylation patterns. The acquisition of a new glycosylation site at HA-160 in H3N2, for instance, is a well-documented strategy for shielding antigenic sites from antibody recognition, according to recent virological research [34].

Our mutational analysis focused on the HA and NA surface glycoproteins, which are the principal targets of the humoral immune response and the components of current vaccines. However, we acknowledge that influenza antigenicity and evolution are whole-genome phenomena, with internal proteins like nucleoprotein (NP) and matrix protein (M1) also contributing to T-cell immunity [35,36,37,38].

Similarly, the remodeling of glycan shields on both HA and NA proteins suggests ongoing selection pressure to evade humoral immunity, which may have significant implications for vaccine strain selection and efficacy.

The identification of NA mutations associated with reduced drug susceptibility, particularly the established permissive mutation E382G in H1N1, as indicated by previous surveillance data [39] and the antibody-evading S245N/S247T double mutation in H3N2, raises important concerns for clinical management, as shown by earlier research [40]. Although phenotypic resistance was not confirmed in this study, the presence of these polymorphisms in circulating strains highlights the critical need for proactive surveillance of antiviral resistance. This ensures that treatment guidelines remain effective and informs the development of next-generation antivirals.

Our study has limitations. The relatively small number of sequenced genomes and the focus on a single geographic region limit the generalizability of our findings. Furthermore, the antigenic and functional impacts of the identified mutations require validation through neutralization assays and reverse genetics. Nevertheless, this genomic snapshot during a unique epidemiological period provides a critical baseline. It emphasizes that influenza virus evolution did not stall during the pandemic but continued on a trajectory that must be closely monitored. Future efforts should integrate phenotypic assays and expand longitudinal sampling to fully assess the public health threat posed by these evolving strains.

## 5. Conclusions

In conclusion, our integrated study demonstrates that despite a dramatic suppression of influenza activity in children in Wuhan during the COVID-19 pandemic, the H1N1 and H3N2 that persisted were genetically active and evolving. The co-circulation of globally sourced clades, coupled with reassortment events and the accumulation of mutations affecting antigenicity and potential drug susceptibility, paints a picture of unceasing viral adaptation. These findings serve as a critical alert that a transient epidemiological trough does not equate to an evolutionary standstill. As influenza circulation returns to pre-pandemic patterns, our data underscore the non-negotiable need for sustained and vigilant genomic surveillance, timely vaccine updates that account for these antigenic changes, and proactive monitoring of antiviral resistance to safeguard public health against the enduring threat of seasonal influenza.

## Figures and Tables

**Table 1:** Demographic and clinical data of inpatients for all IAV virus subtypes during the duration of the research.

Variable	H1N1 *N* = 57	H3N2 *N* = 18	Untypeable *N* = 34
Sex			
Male	35 (61.40%)	8 (44.44%)	15 (44.11%)
Female	22 (38.59%)	10 (55.55%)	19 (55.88%)
Age (years)			
<1 year old (Baby)	12 (21.05%)	1 (5.55%)	5 (14.70%)
1–3 years old (Infant)	11 (19.29%)	2 (11.11%)	4 (11.76%)
3–6 years old (Pre-school children)	25 (43.85%)	9 (50%)	10 (29.41%)
>6 years old (School-age children)	9 (15.78%)	6 (33.33%)	15 (44.11%)
Co-infection			
Bacteria	13 (22.80%)	4 (22.22%)	6 (17.64%)
Virus	3 (5.26%)	1 (5.55%)	2 (5.88%)
M. pneumonia		1 (5.55%)	2 (5.88%)
Symptoms			
Fever	51 (89.47%)	18 (100%)	34 (100%)
Cough	54 (94.73%)	18 (100%)	33 (97.05%)
Wheezing	13 (22.80%)	2 (11.11%)	5 (14.70%)
Vomiting	6 (10.52%)	2 (11.11%)	8 (23.52%)
Diarrhea	1 (1.75%)		2 (5.88%)
Muscle soreness	1 (1.75%)		2 (5.88%)
Headache	2 (5.88%)		4 (11.76%)
Abdominal pain	4 (7.01%)	3 (16.66%)	4 (11.76%)
Hoarseness	4 (7.01%)	1 (5.55%)	
Complications			
Pneumonia with lung consolidation	4 (7.01%)	2 (11.11%)	1 (2.94%)
Pneumonia	27 (47.37%)	10 (55.56%)	15 (44.12%)
Bronchitis	1 (0.91%)		4 (3.66%)
Myositis			1 (0.91%)
No complication	25 (22.93%)	6 (5.50%)	13 (11.92%)

## Data Availability

The whole-genome sequences generated in this study have been deposited in the Global Initiative on Sharing All Influenza Data (GISAID) EpiFlu database, and the Accession numbers are listed in Table S9.

## Supplementary Materials

The following supporting information can be downloaded at: https://www.mdpi.com/article/10.3390/v18020210/s1. Table S1: Average Sequence Depth & Coverage (A/H1N1). Table S2: Average Sequence Depth & Coverage (A/H3N2). Table S3: Amino acid substitutions in antigenic sites of the HA gene of H1N1 compared with vaccine strain A/Victoria/4897/2022. Table S4: Amino acid substitutions in antigenic sites of the HA gene of H3N2 compared with the vaccine strain A/Hong Kong/4801/2014. Table S5: Amino acid substitutions in the NA gene of influenza virus A(H1N1) compared with vaccine strain A/Victoria/4897/2022. Table S6: Amino acid substitutions in the NA gene of influenza virus A(H3N2) compared with vaccine strain A/Hong Kong/4801/2014. Table S7: N-glycosylation sites of HA and NA gene (A/H1N1). Table S8: N-glycosylation sites of HA and NA gene (A/H3N2). Table S9: Strain names and accession numbers of eight segments of influenza A(H3N2) and A(H1N1pdm09) viruses, collected in Wuhan 2023, sequenced during this study, and deposited in GISAID. Figures S1–S16: Phylogenetic trees of eight segments of the influenza A(H1N1pdm09) and A(H3N2) viruses collected in Wuhan in 2023.

## References

[1] WHO (2025). Influenza (Seasonal)—Fact Sheet.

[2] Sadedini L., Lito G.K., Kuneshka N., Skenderi E., Shkembi A., Shtembari D., Gjika A. (2025). Prevalence of influenza in children presented in pediatric emergency department. Cough.

[3] Martin D., Hönemann M., Liebert U.G. (2021). Dynamics of nosocomial parainfluenza virus type 3 and influenza virus infections at a large German University Hospital between 2012 and 2019. Diagn. Microbiol. Infect. Dis..

[4] Tsai C.F., Liu Y.C., Chang T.H., Wu E.T., Chang L. (2023). The clinical predictors of and vaccine protection against severe influenza infection in children. J. Med. Virol..

[5] Watanabe S., Hoshina T., Kojiro M., Kusuhara K. (2021). The recent characteristics of influenza-related hospitalization in Japanese children. Eur. J. Clin. Microbiol. Infect. Dis..

[6] Frankl S., Coffin S.E., Harrison J.B., Swami S.K., McGuire J.L. (2021). Influenza-associated neurologic complications in hospitalized children. J. Pediatr..

[7] Fu C., Huang Q., Zhao J., Mo L., Tang W., Lu J., Zhang Y., Lu X., Huang Y., Feng Y. (2025). Clinical characteristics and co-infection analysis of influenza a virus in pediatric respiratory infections: A study based on tNGS technology. Eur. J. Clin. Microbiol. Infect. Dis..

[8] Uchida M., Yamauchi T. (2022). Rate of diagnosed seasonal influenza in children with influenza-like illness: A cross-sectional study. PLoS ONE.

[9] Hoy G., Maier H.E., Kuan G., Sánchez N., López R., Meyers A., Plazaola M., Ojeda S., Balmaseda A., Gordon A. (2023). Increased influenza severity in children in the wake of SARS-CoV-2. Influ. Other Respir. Viruses.

[10] CDC (2024). How Flu Viruses Can Change: ‘Drift’ and ‘Shift’.

[11] Kim H., Webster R.G., Webby R.J. (2018). Influenza virus: Dealing with a drifting and shifting pathogen. Viral Immunol..

[12] Wang Q. (2010). Influenza antigenic drift: What is the driving force?. Cellscience.

[13] Tankeshwar A. (2013). Antigenic Drift and Antigenic Shift in Influenza Virus. Microbe Online.

[14] Carrat F., Flahault A. (2007). Influenza vaccine: The challenge of antigenic drift. Vaccine.

[15] Feng L., Zhang T., Wang Q., Xie Y., Peng Z., Zheng J., Qin Y., Zhang M., Lai S., Wang D. (2021). Impact of COVID-19 outbreaks and interventions on influenza in China and the United States. Nat. Commun..

[16] Chen L.-J., Guo J.-J., Guo W.-W., Shen E.-X., Wang X., Li K.-J., Yan J., Shi M., Li Y.-R., Hou W. (2020). Molecular epidemiology and vaccine compatibility analysis of seasonal influenza viruses in Wuhan, 2016–2019. Virol. Sin..

[17] Zeng H., Cai M., Li S., Chen X., Xu X., Xie W., Xiong Y., Long X. (2023). Epidemiological characteristics of seasonal influenza under implementation of zero-COVID-19 strategy in China. J. Infect. Public Health.

[18] Chen L., Guo Y., López-Güell K., Ma J., Dong Y., Xie J., Alhambra D.P. (2025). Immunity Debt for Seasonal Influenza After the COVID-19 Pandemic and as a Result of Nonpharmaceutical Interventions: An Ecological Analysis and Cohort Study. Adv. Sci..

[19] Asante I.A., Nyarko S.O., Awuku-Larbi Y., Obeng R.A., Sarpong G.M., Amenuvor E.A.A., Adusei-Poku M., Boatemaa L., Magnusen V., Wutsika J. (2024). Decreased influenza activity during the COVID-19 pandemic in Ghana, 2020. Front. Public Health.

[20] Liu X., Peng Y., Chen Z., Jiang F., Ni F., Tang Z., Yang X., Song C., Yuan M., Tao Z. (2024). Correction: Impact of non-pharmaceutical interventions during COVID-19 on future influenza trends in Mainland China. BMC Infect. Dis..

[21] Chow E.J., Uyeki T.M., Chu H.Y. (2023). The effects of the COVID-19 pandemic on community respiratory virus activity. Nat. Rev. Microbiol..

[22] Yu X., Xu C., Huang W., Xu X., Xie W., Long X. (2021). The incidence of influenza in children was decreased in the first flu season after COVID-19 pandemic in Wuhan. J. Infect. Public Health.

[23] Fitzner J., Qasmieh S., Mounts A.W., Alexander B., Besselaar T., Briand S., Brown C., Clark S., Dueger E., Gross D. (2018). Revision of clinical case definitions: Influenza-like illness and severe acute respiratory infection. Bull. World Health Organ..

[24] Terrier O., Josset L., Textoris J., Marcel V., Cartet G., Ferraris O., N’guyen C., Lina B., Diaz J.-J., Bourdon J.-C. (2011). Cellular transcriptional profiling in human lung epithelial cells infected by different subtypes of influenza A viruses reveals an overall down-regulation of the host p53 pathway. Virol. J..

[25] Zhao X.-N., Zhang H.-J., Li D., Zhou J.-N., Chen Y.-Y., Sun Y.-H., Adeola A.C., Fu X.-Q., Shao Y., Zhang M.-L. (2020). Whole-genome sequencing reveals origin and evolution of influenza A (H1N1) pdm09 viruses in Lincang, China, from 2014 to 2018. PLoS ONE.

[26] Li L., Changrob S., Fu Y., Stovicek O., Guthmiller J.J., McGrath J.J., Dugan H.L., Stamper C.T., Zheng N.-Y., Huang M. (2022). Librator: A platform for the optimized analysis, design, and expression of mutable influenza viral antigens. Brief. Bioinform..

[27] Yang Z., Zhan Y., Li Z., Lin Z., Fang Z., Li H., Chen X., Ding B., Zeng H., Zhang X. (2025). Efficacy and safety of onradivir in adults with acute uncomplicated influenza A infection in China: A multicentre, double-blind, randomised, placebo-controlled and oseltamivir-controlled, phase 3 trial. Lancet Respir. Med..

[28] Ge X., Chen Y., Wu W., Lu J., Wang Y., Li Z. (2024). Safety and effectiveness of baloxavir marboxil and oseltamivir for influenza in children: A real-world retrospective study in China. Front. Pediatr..

[29] Li X., Liu B., Duan N., Xiong Y., Zhang Y., Zhang C., Lu C., Li L. (2022). Clinical efficacy and safety of Chinese patent medicine combined with oseltamivir for the treatment of adult influenza: A systematic review and meta-analysis. Am. J. Chin. Med..

[30] Jiang M., Jia M., Wang Q., Sun Y., Xu Y., Dai P., Yang W., Feng L. (2024). Changes in the Epidemiological Features of Influenza After the COVID-19 Pandemic in China, the United States, and Australia: Updated Surveillance Data for Influenza Activity. Interact. J. Med. Res..

[31] Domingo E., Martínez-González B., Somovilla P., García-Crespo C., Soria M.E., de Ávila A.I., Gadea I., Perales C. (2025). A general and biomedical perspective of viral quasispecies. RNA.

[32] WHO (2024). Recommended Composition of Influenza Virus Vaccines for Use in the 2024–2025 Northern Hemisphere Influenza Season.

[33] Bedford T., Riley S., Barr I.G., Broor S., Chadha M., Cox N.J., Daniels R.S., Gunasekaran C.P., Hurt A.C., Kelso A. (2015). Global circulation patterns of seasonal influenza viruses vary with antigenic drift. Nature.

[34] Gong Y.-N., Tsao K.-C., Chen G.-W. (2019). Inferring the global phylodynamics of influenza A/H3N2 viruses in Taiwan. J. Formos. Med. Assoc..

[35] Rak A., Isakova-Sivak I., Rudenko L. (2023). Nucleoprotein as a promising antigen for broadly protective influenza vaccines. Vaccines.

[36] Vatzia E., Paudyal B., Dema B., Carr B.V., Sedaghat-Rostami E., Gubbins S., Sharma B., Moorhouse E., Morris S., Ulaszewska M. (2024). Aerosol immunization with influenza matrix, nucleoprotein, or both prevents lung disease in pig. npj Vaccines.

[37] Mosmann T.R., McMichael A.J., LeVert A., McCauley J.W., Almond J.W. (2024). Opportunities and challenges for T cell-based influenza vaccines. Nat. Rev. Immunol..

[38] Zhu Y., Sun Y., Deng X., Cao P., Li S., Yu H., Sheng S., Cong Y. (2025). Matrix protein 1 (M1) of influenza A virus: Structural and functional insights. Emerg. Microbes Infect..

[39] Tramuto F., Maida C.M., Randazzo G., Previti A., Sferlazza G., Graziano G., Costantino C., Mazzucco W., Vitale F. (2024). Insights into Genetic and Antigenic Characteristics of Influenza A (H1N1) pdm09 Viruses Circulating in Sicily During the Surveillance Season 2023–2024: The Potential Effect on the Seasonal Vaccine Effectiveness. Viruses.

[40] Wan H., Gao J., Yang H., Yang S., Harvey R., Chen Y.-Q., Zheng N.-Y., Chang J., Carney P.J., Li X. (2019). The neuraminidase of A (H3N2) influenza viruses circulating since 2016 is antigenically distinct from the A/Hong Kong/4801/2014 vaccine strain. Nat. Microbiol..

