# Supplementary material for: Genomic Evolution of Influenza A(H1N1)pdm09 and A/H3N2 Viruses Among Children in Wuhan, China, Spanning the COVID-19 Pandemic (2020–2023)
Source: Viruses. 2026 Feb 5;18(2):210. doi: 10.3390/v18020210 (PMC12945282; doi:10.3390/v18020210)

Supplementary Figure S1

HA H1N1

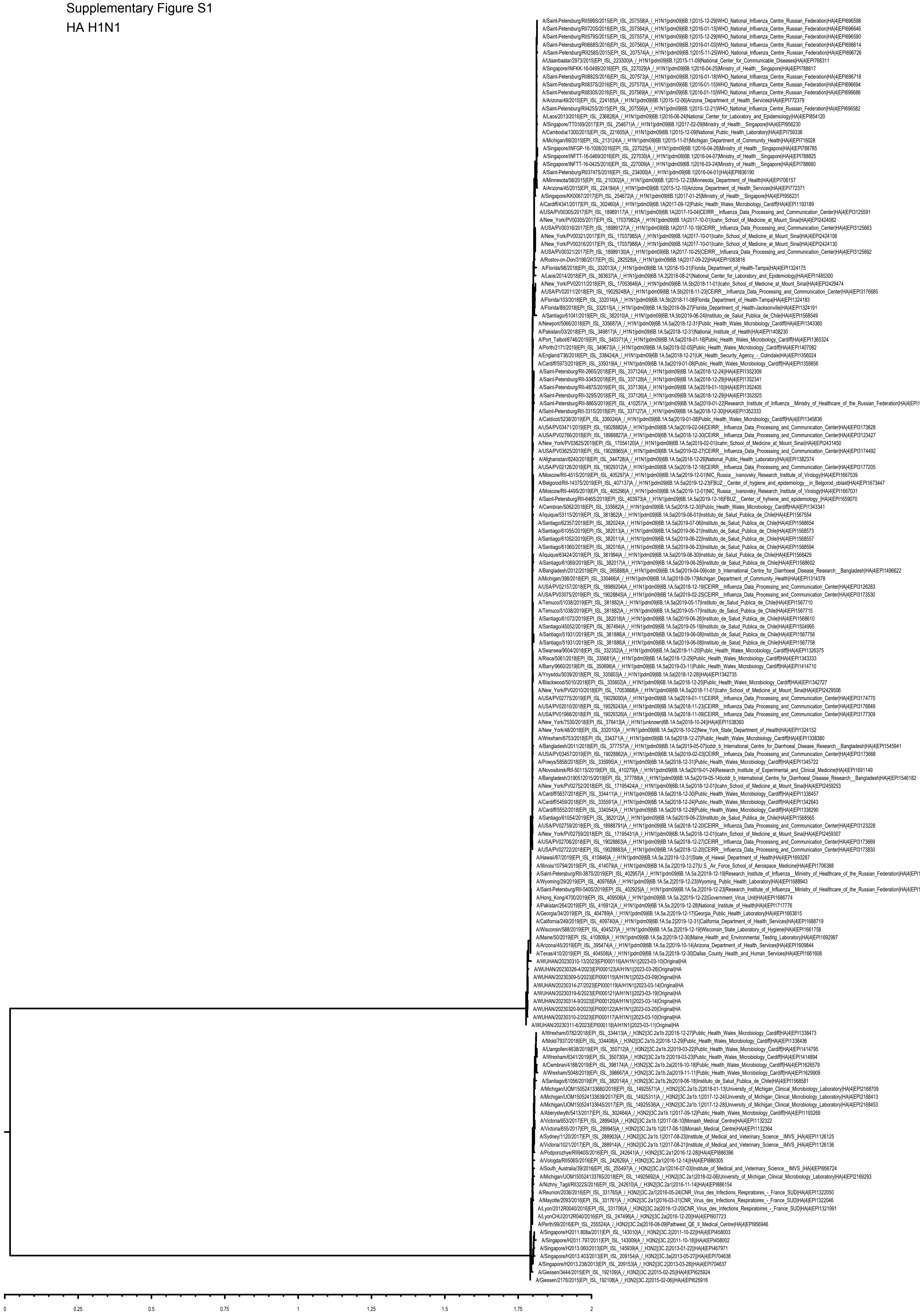

Supplementary Figure S2

NA H1N1

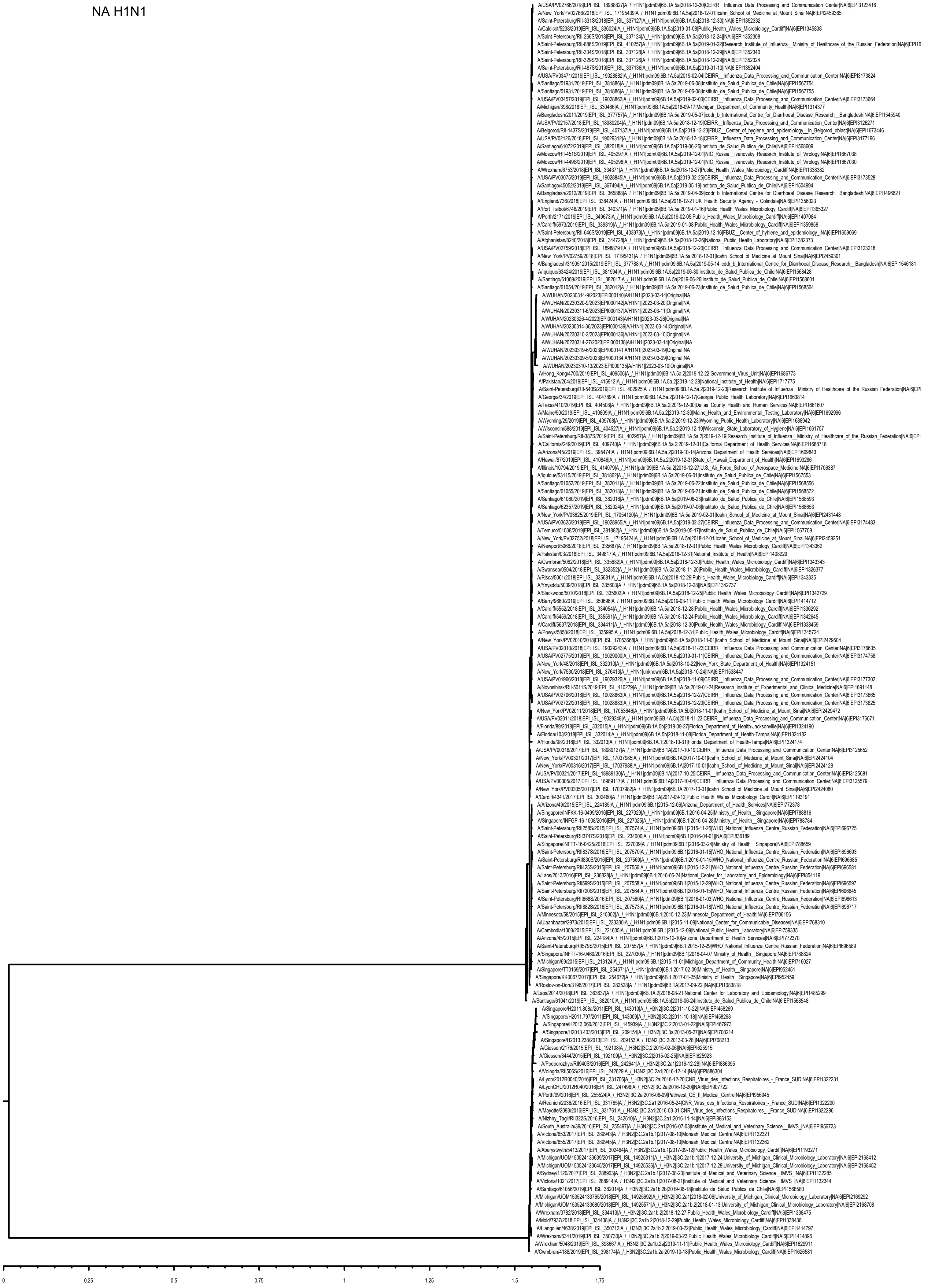

Supplementary Figure S3

PB2 H1N1

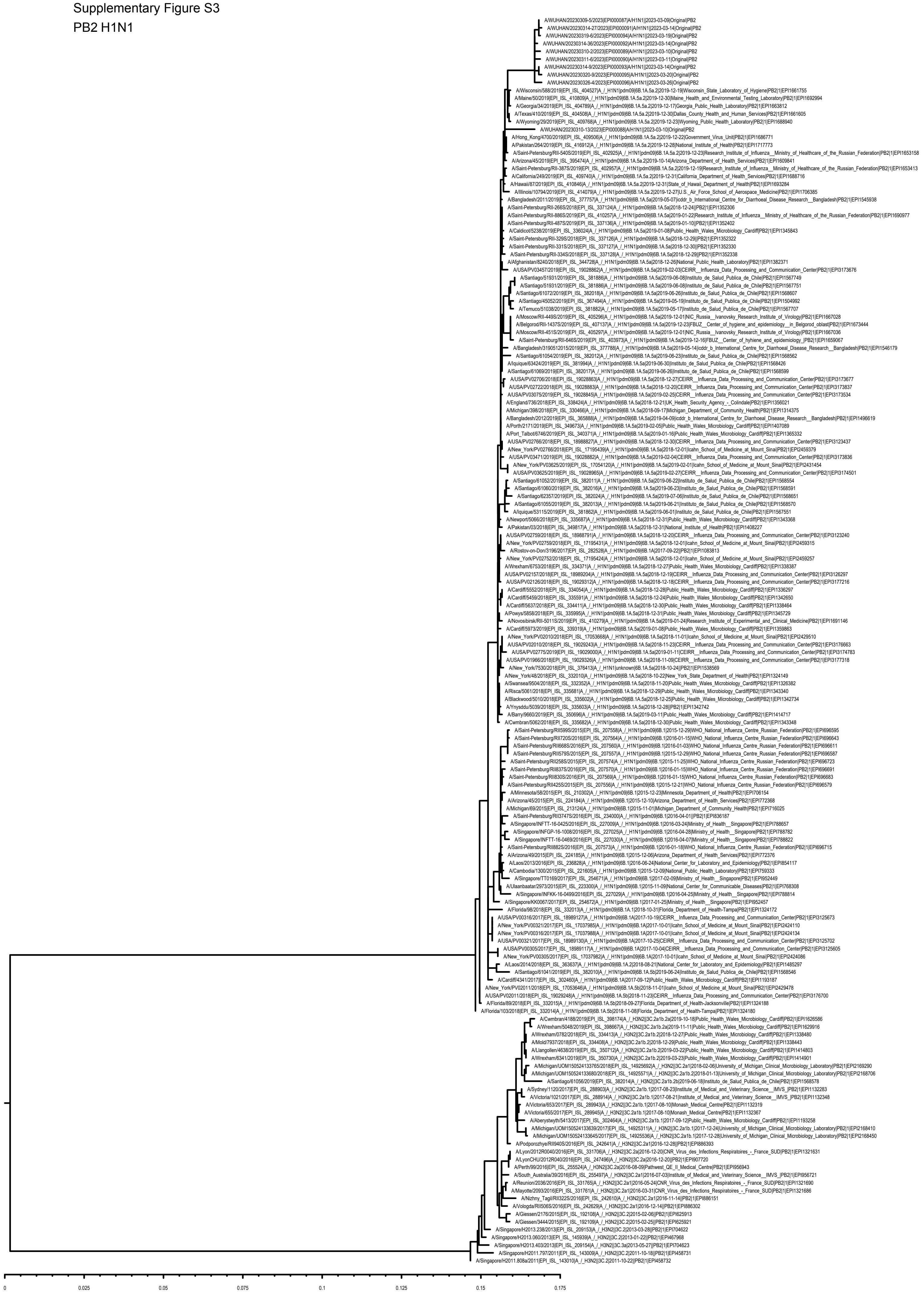

Supplementary Figure S4

PB1 H1N1

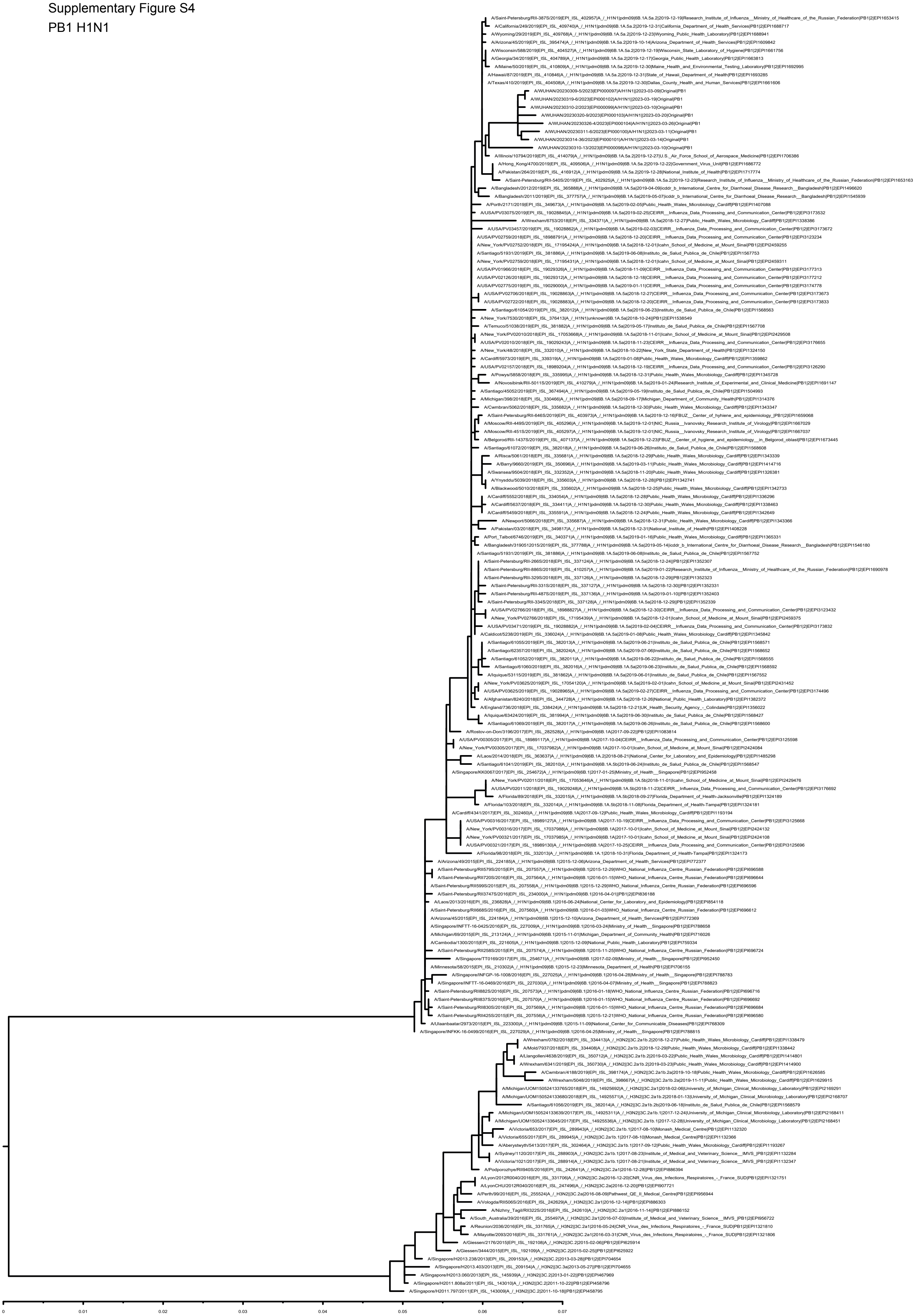

Supplementary Figure S5

PA H1N1

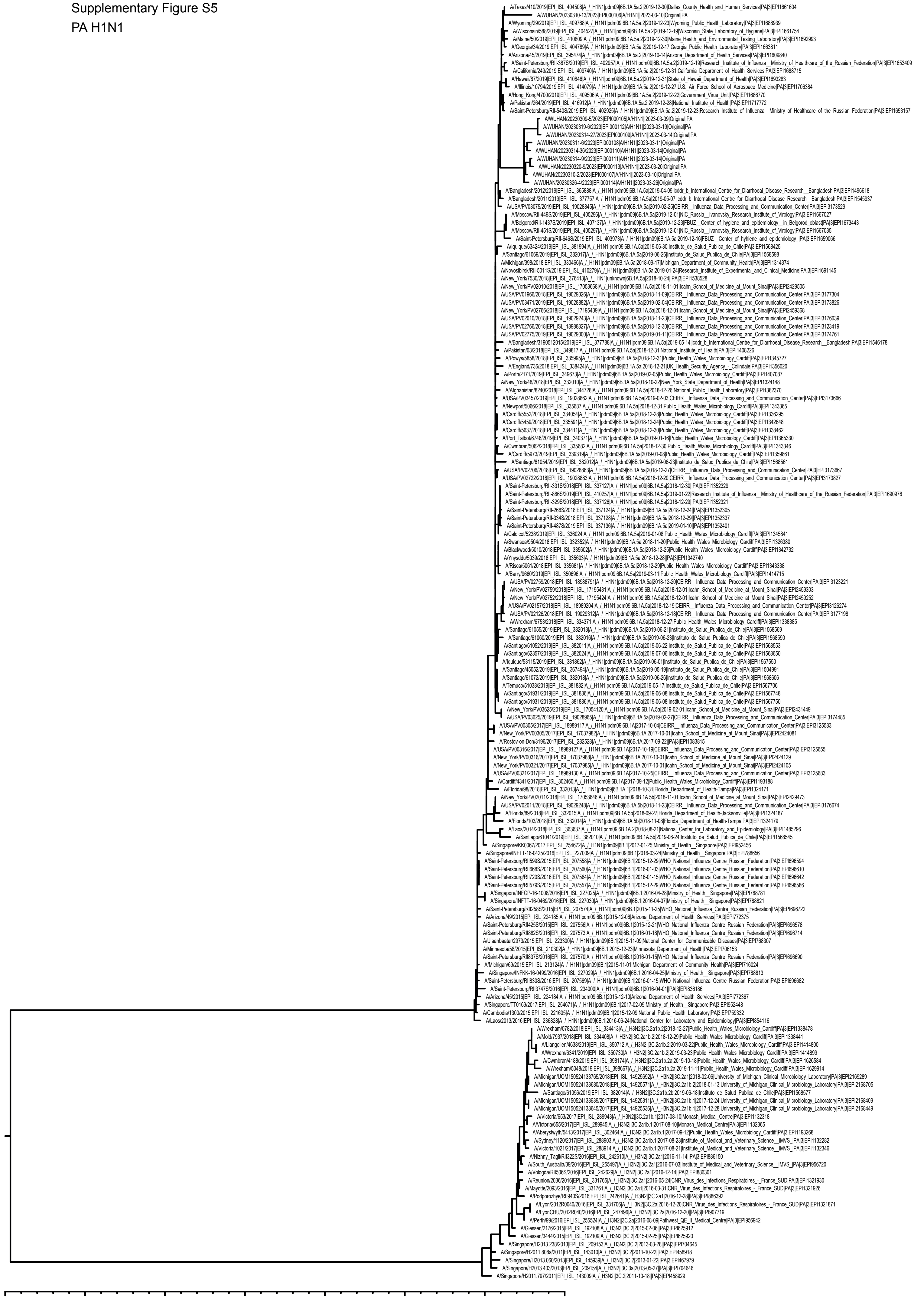

Supplementary Figure S6

NP H1N1

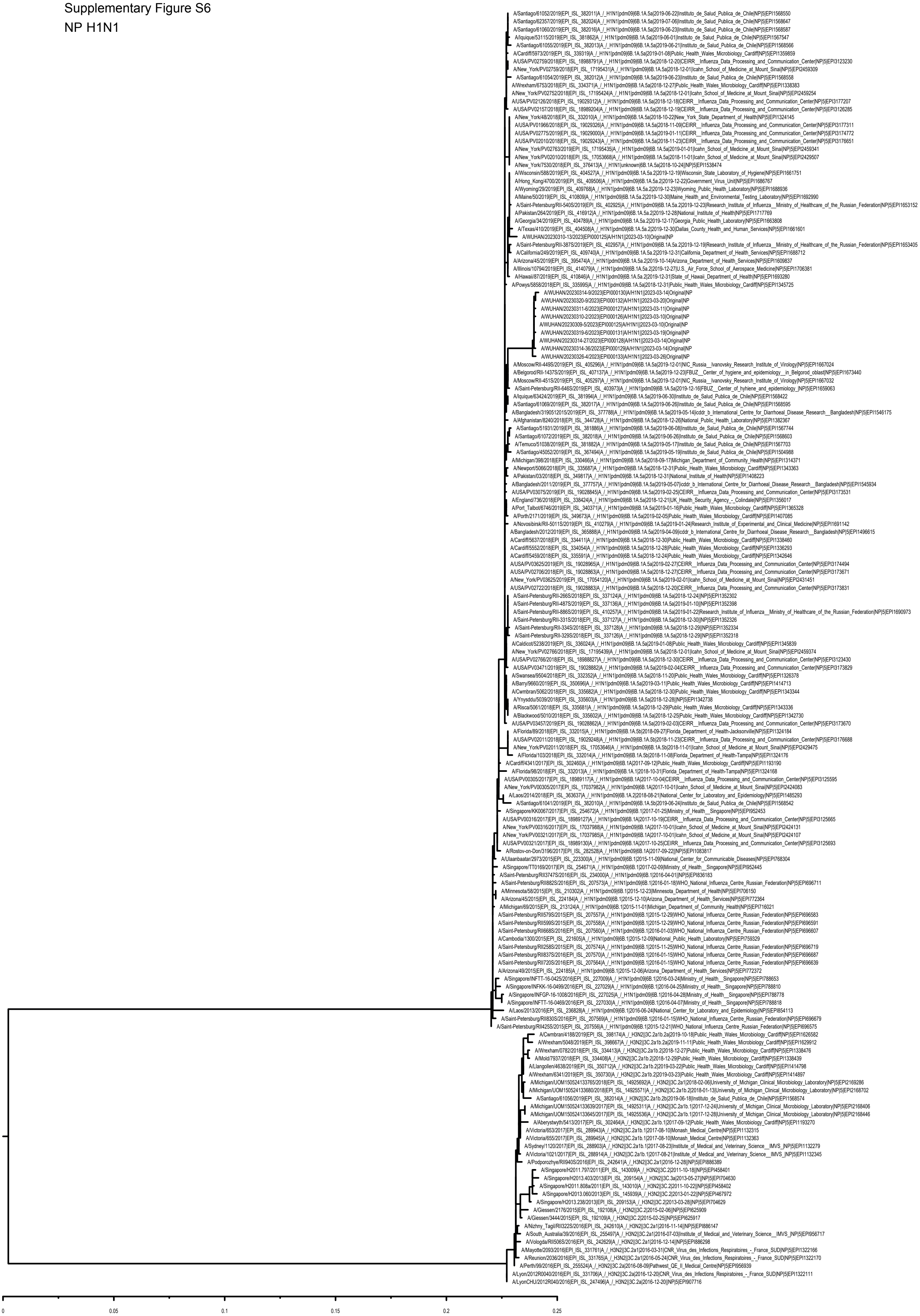

Supplementary Figure S7

MP H1N1

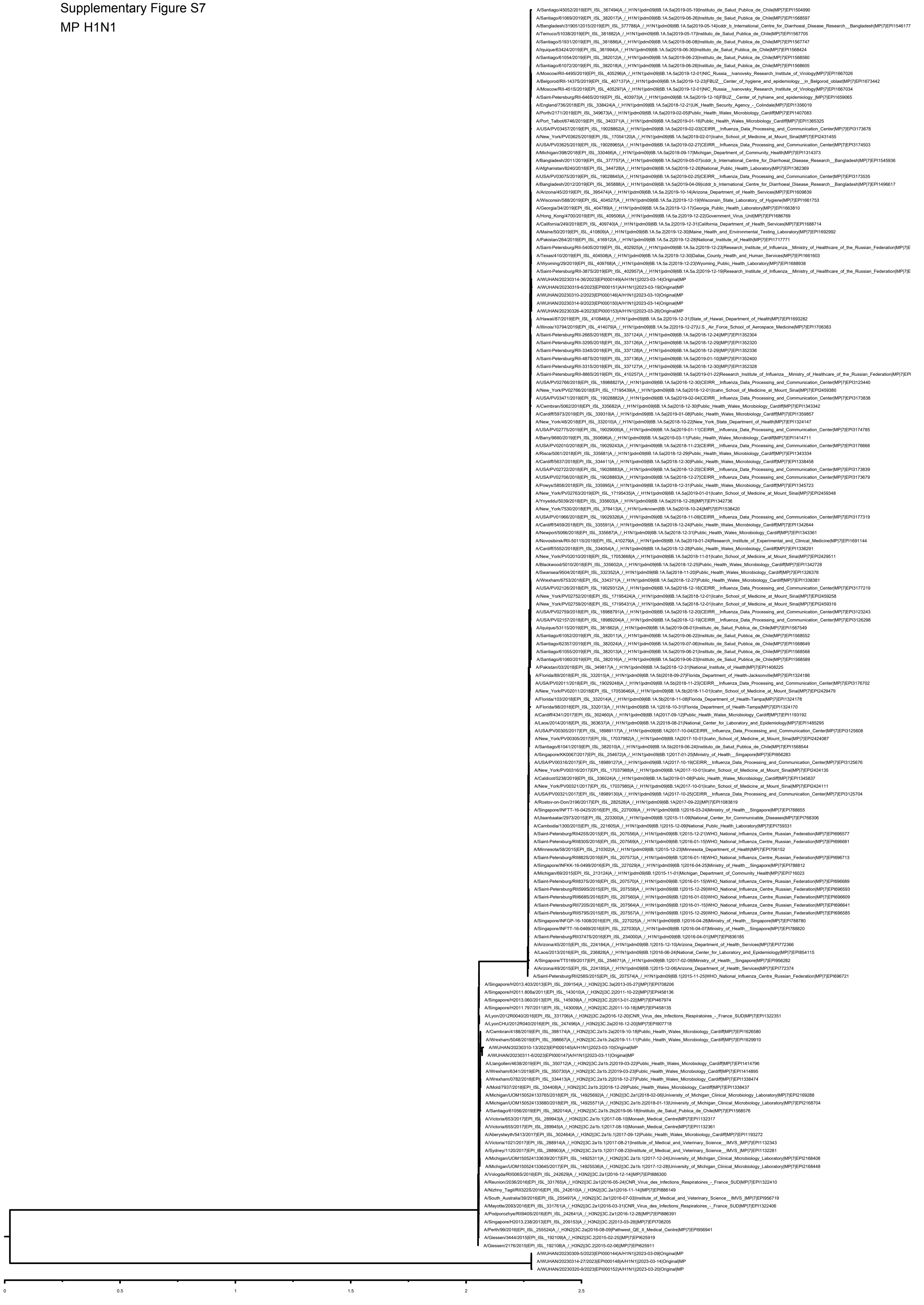

Supplementary Figure S8

NS H1N1

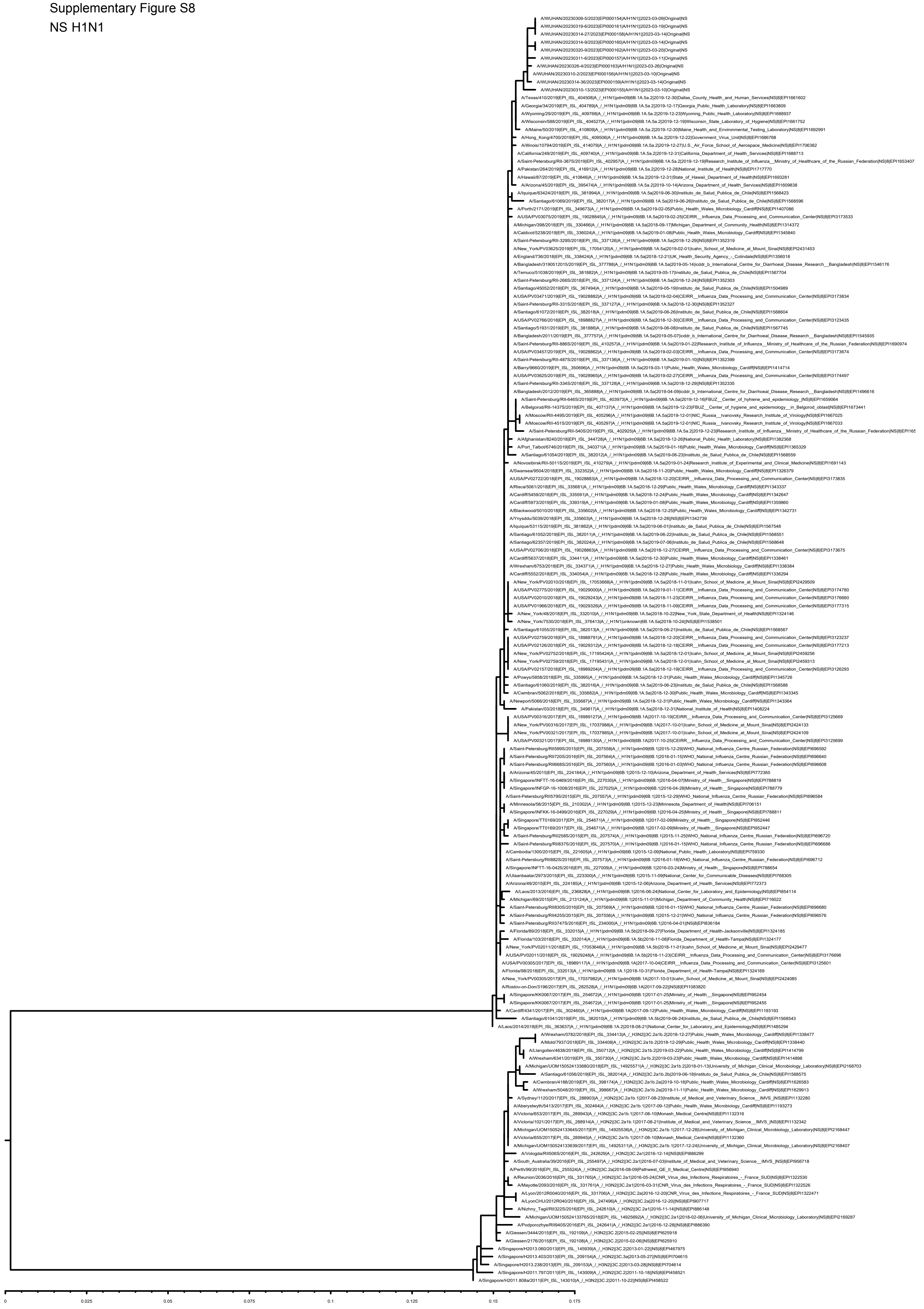

Supplementary Figure S9

HA H3N2

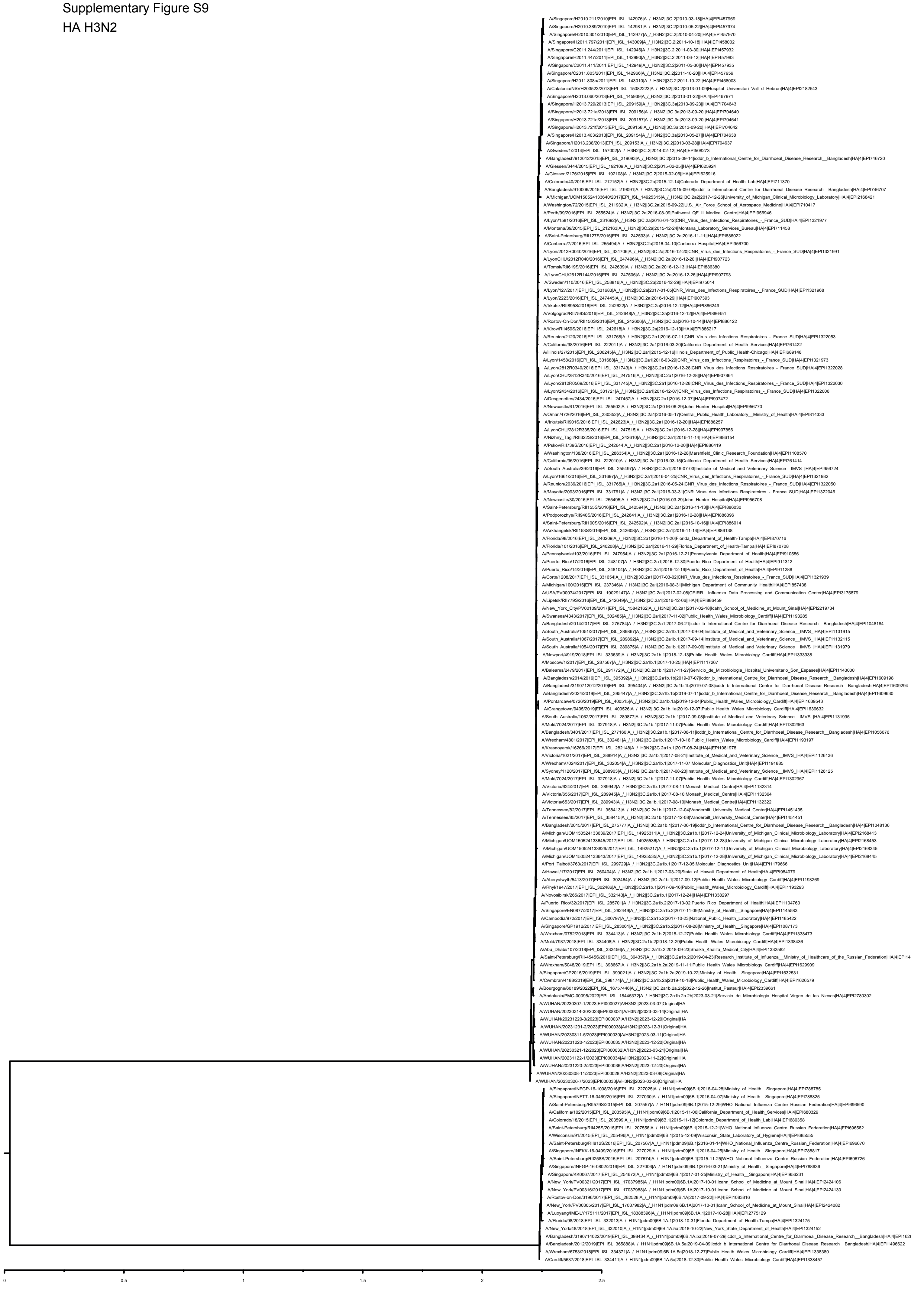

Supplementary Figure S10

NA H3N2

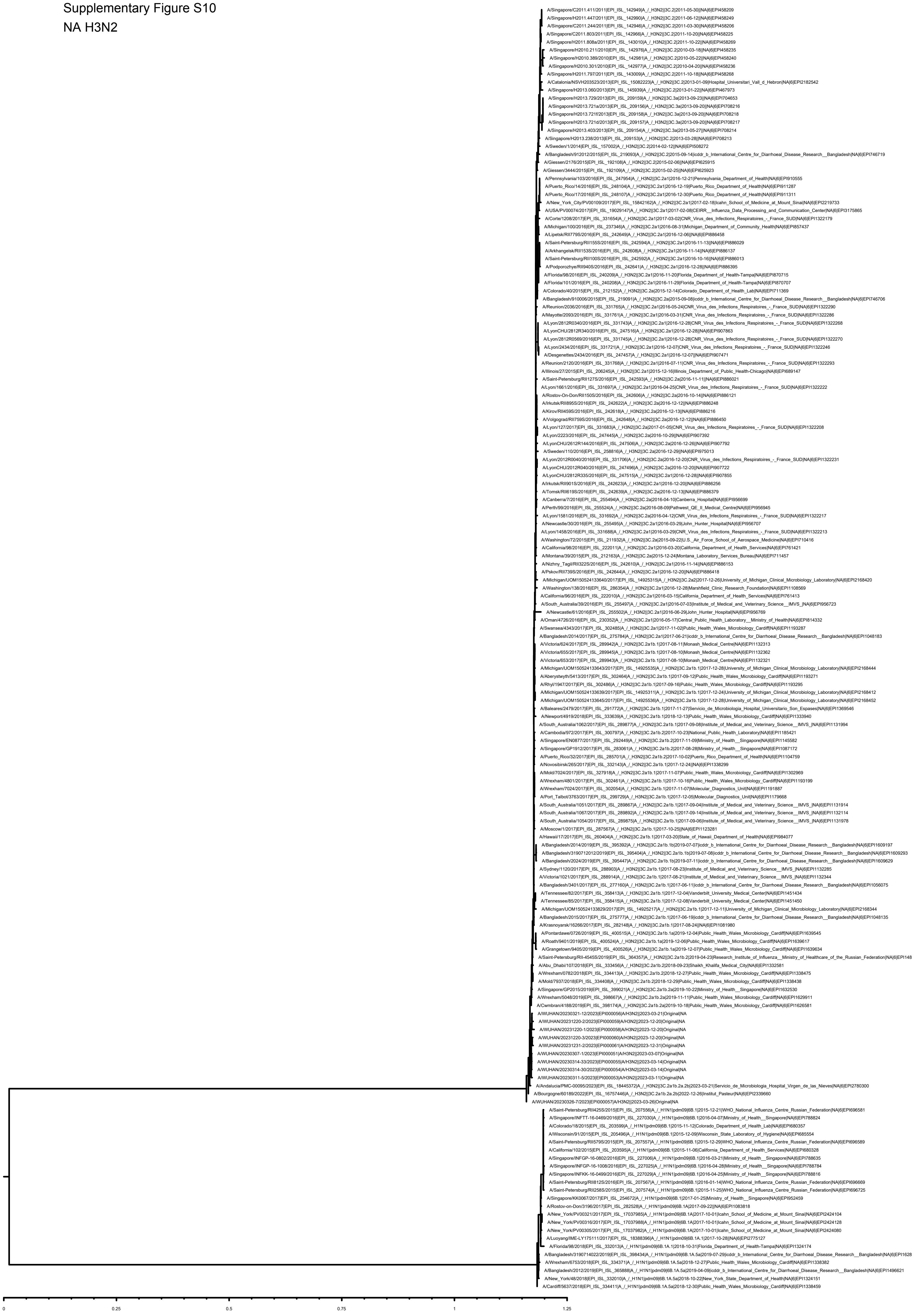

Supplementary Figure S11

PB2 H3N2

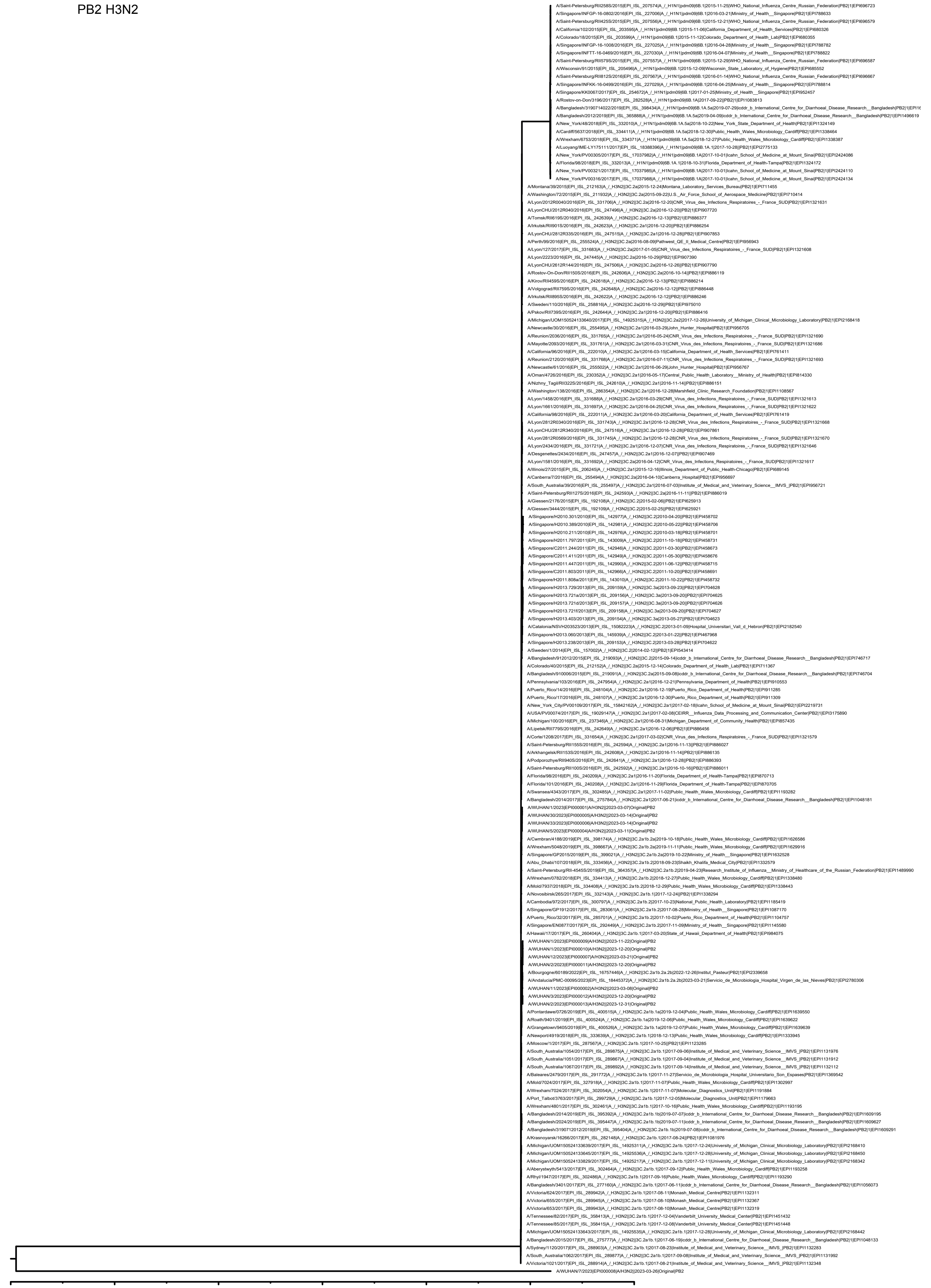

Supplementary Figure S12

PB1 H3N2

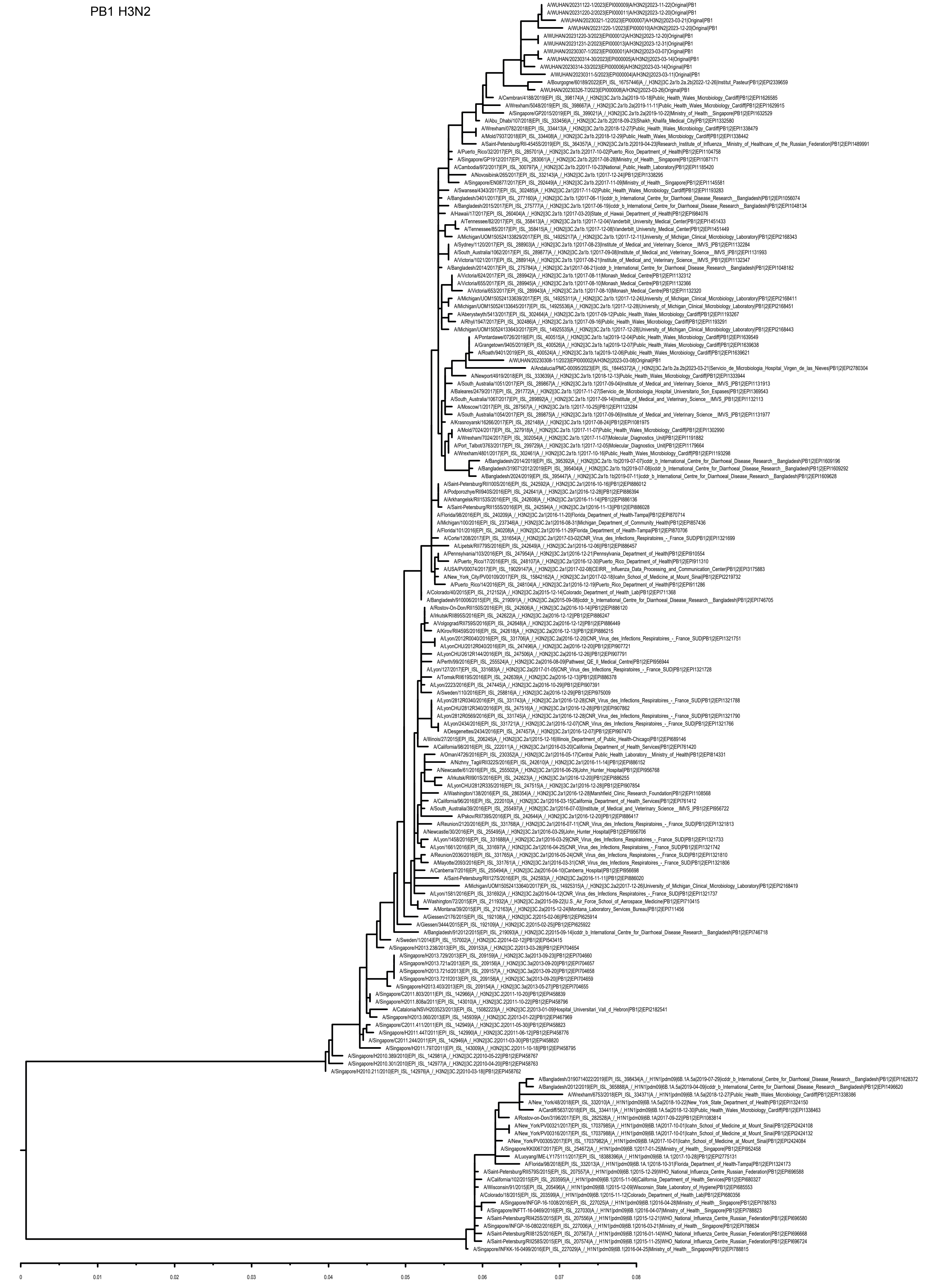

Supplementary Figure S13

PA H3N2

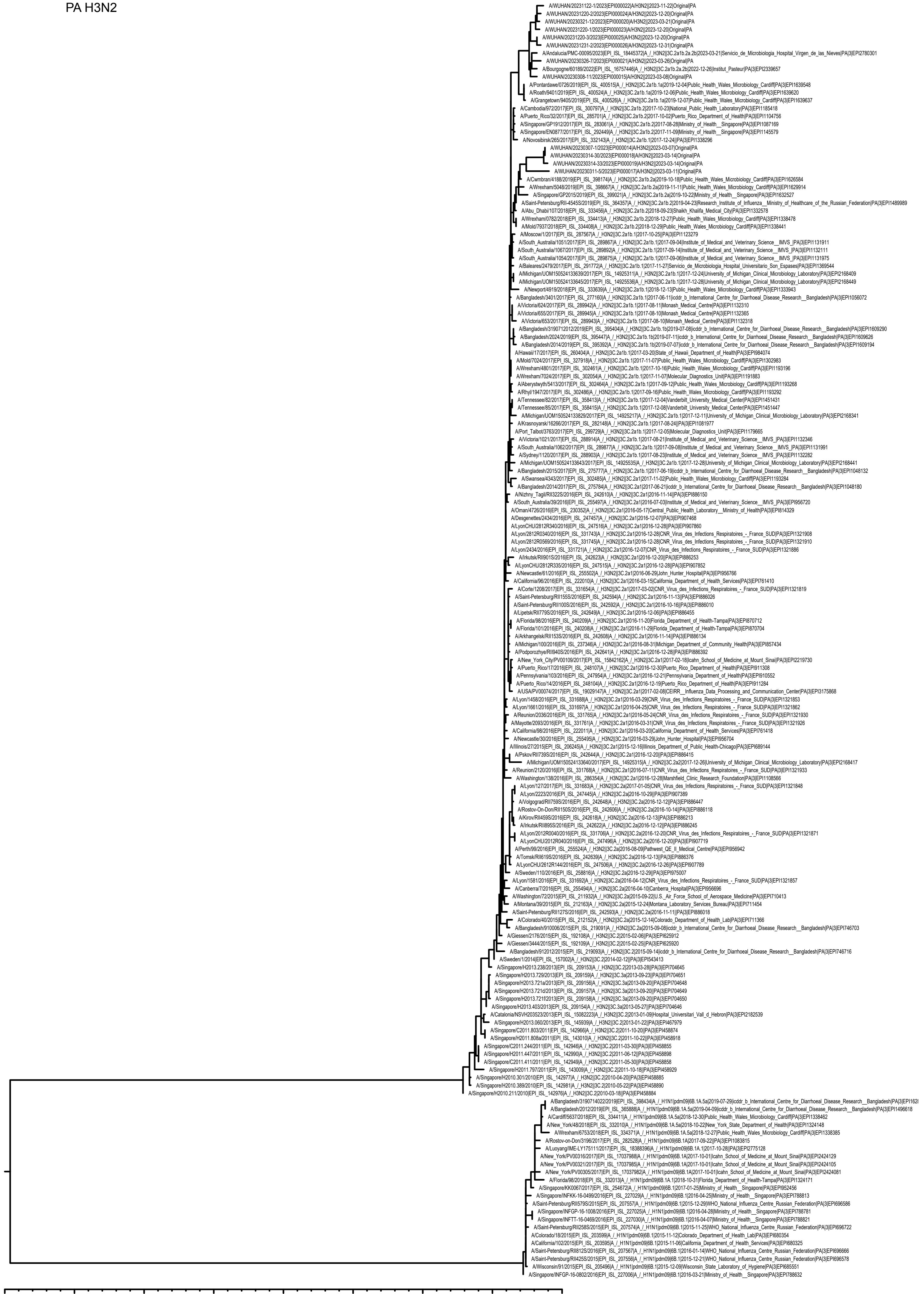

Supplementary Figure S14

NP H3N2

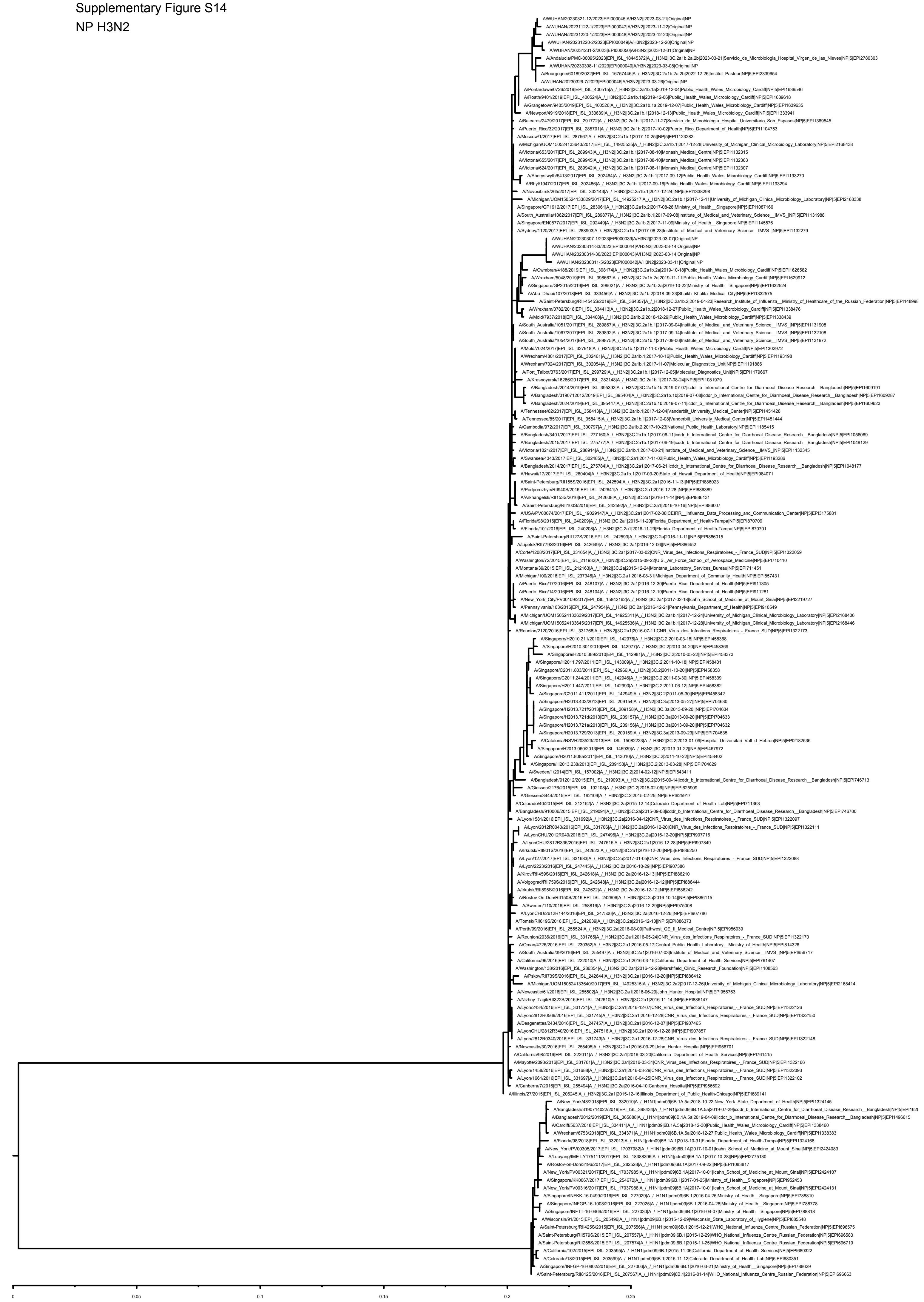

Supplementary Figure S15

MP H3N2

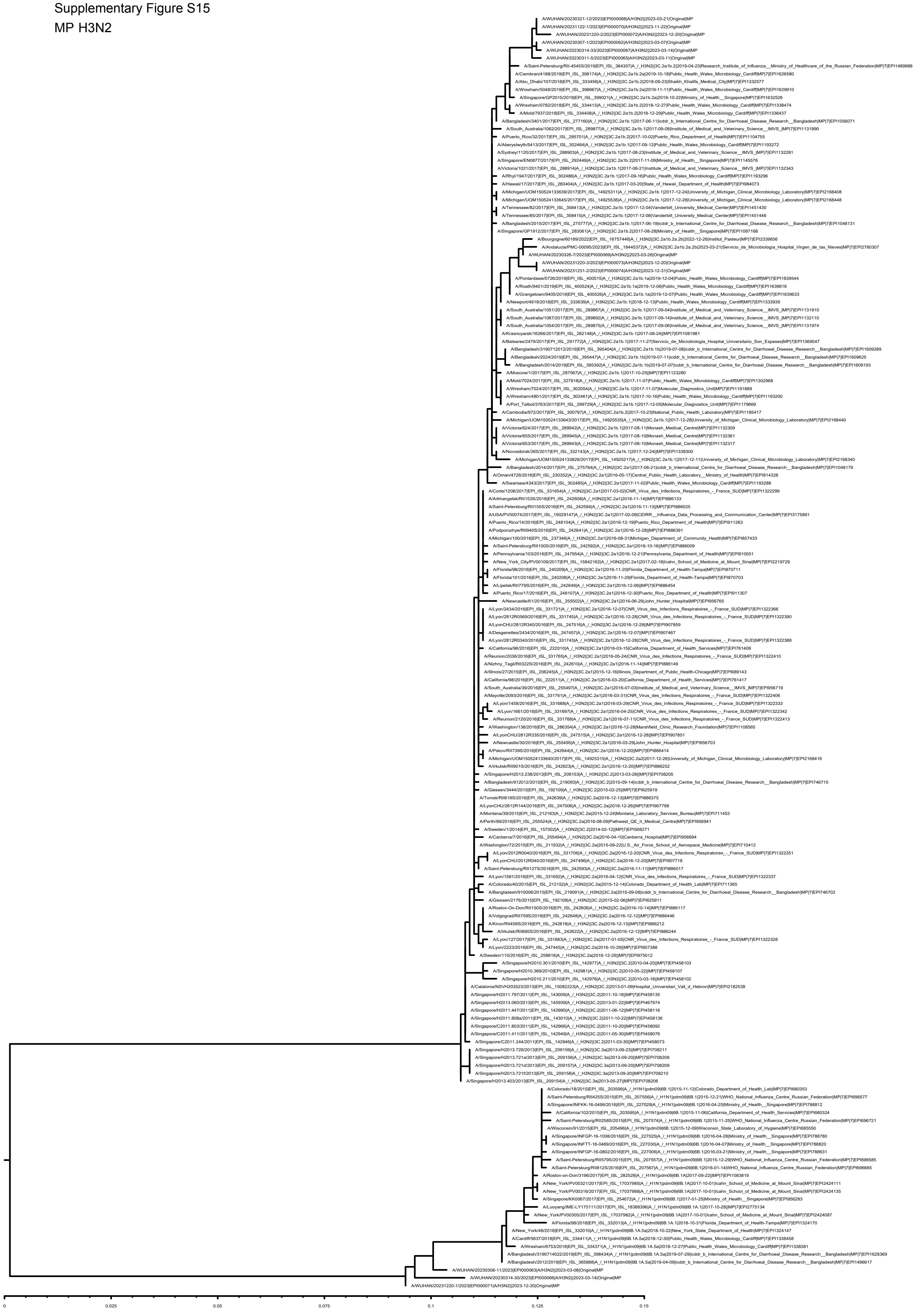

Supplementary Figure S16

NS H3N2

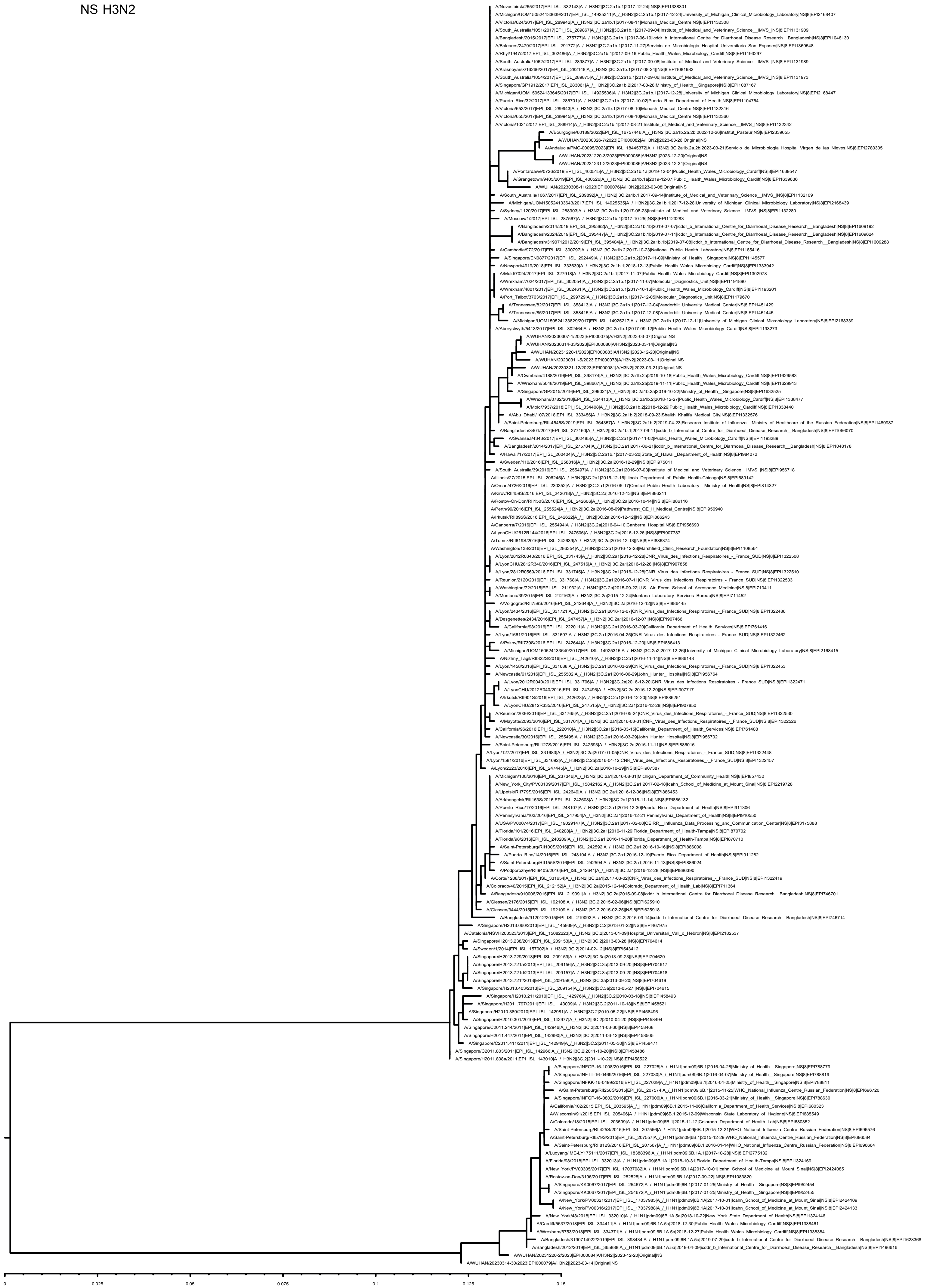

Supplement: Supplementary file 1 [file viruses-18-00210-s001.zip › Supplementary_Figures_S1-S16.pdf]
