# Supplementary material for: Genomic Evolution of Influenza A(H1N1)pdm09 and A/H3N2 Viruses Among Children in Wuhan, China, Spanning the COVID-19 Pandemic (2020–2023)
Source: Viruses. 2026 Feb 5;18(2):210. doi: 10.3390/v18020210 (PMC12945282; doi:10.3390/v18020210)
Supplement: Supplementary file 1 [file viruses-18-00210-s001.zip › Supplementary_Tables_S1-S8.pdf]

Supplementary Table S1. Average Sequence Depth & Coverage (A/H1N1).

Sample	Accession	PB2	PB1	PA	HA	NP	NA	MP	NS
A/WH/20230309-5/2023|A/H1N1	EPI_ISL_20239865	36636.9 (96.3%)	25802.9 (98.7%)	8713.74 (98.8%)	44159.5 (98.7%)	12030.4 (98.6%)	35149.3 (98.7%)	98234.7 (98.7%)	67432.4 (98%)
A/WH/20230310-13/2023|A/H1N1	EPI_ISL_20239867	151479 (98.7%)	101779 (98.7%)	111963 (98.8%)	419305 (98.7%)	198022 (98.8%)	444963 (98.7%)	242091 (98.8%)	1099800 (98.7%)
A/WH/20230310-2/2023|A/H1N1	EPI_ISL_20239866	231585 (98.8%)	213781 (98.7%)	122665 (98.8%)	386007 (98.7%)	262607 (98.8%)	277000 (98.7%)	1102700 (98.6%)	774510 (98.6%)
A/WH/20230311-6/2023|A/H1N1	EPI_ISL_20239868	252124 (98.7%)	145928 (98.7%)	153057 (98.8%)	364957 (98.7%)	150453 (98.7%)	319288 (98.7%)	141198 (98.8%)	793907 (98.7%)
A/WH/20230314-27/2023|A/H1N1	EPI_ISL_20239961	276808 (96.7%)	0	3502.17 (98.8%)	185950 (96.6%)	1595.43 (98.7%)	88355.8 (95%)	511441 (98.7%)	166714 (98.5%)
A/WH/20230314-36/2023|A/H1N1	EPI_ISL_20239962	193441 (98.7%)	43584.1 (98.7%)	98424.1 (98.8%)	0	45983 (98.5%)	152250 (98.7%)	168236 (97.5%)	197011 (98.4%)
A/WH/20230314-9/2023|A/H1N1	EPI_ISL_20239960	260826 (98.7%)	0	117703 (98.7%)	932663 (98.7%)	27858.6 (98.8%)	1102570 (98.7%)	915907 (98.8%)	205693 (98.7%)
A/WH/20230319-6/2023|A/H1N1	EPI_ISL_20239963	9878.93 (98.7%)	592.236 (98.7%)	7976.28 (98.8%)	13495.7 (98.7%)	5390.55 (98.8%)	15762.2 (95%)	37291.6 (92.6%)	31786.9 (98.7%)
A/WH/20230320-9/2023|A/H1N1	EPI_ISL_20239964	127638 (98.5%)	36703.5 (98.5%)	49202.3 (98.6%)	159736 (98.7%)	23725.6 (94.7%)	197226 (98.6%)	509836 (98.7%)	330276 (91.2%)
A/WH/20230326-4/2023|A/H1N1	EPI_ISL_20239960	884.852 (98.5%)	4547.23 (98.7%)	2866.84 (98.8%)	2038.69 (98.7%)	3844.05 (98.8%)	1056.43 (94.6%)	10343.8 (82%)	15152.9 (98.6%)

Supplementary Table S2. Average Sequence Depth & Coverage (A/H3N2).

**Sample**	Accession	**PB2**	**PB1**	**PA**	**HA**	**NP**	**NA**	**MP**	**NS**
A/WH/20230307-1/2023|A/H3N2	EPI_ISL_20238018	223700 (98.6%)	69129.6 (98.8%)	174329 (98.8%)	310454 (98.7%)	214459 (98.8%)	295739 (98.7%)	1504550 (98.8%)	228059 (98.7%)
A/WH/20230308-11/2023|A/H3N2	EPI_ISL_20238019	136901 (94.8%)	82182.3 (93%)	154895 (98.7%)	110871 (98.7%)	24699 (98.4%)	0	45748.3 (98.6%)	15952.3 (98.8%)
A/WH/20230311-5/2023|A/H3N2	EPI_ISL_20239487	200491 (98.6%)	102591 (98.5%)	550768 (98.8%)	296773 (98.6%)	270247 (98.8%)	180753 (98.7%)	1159510 (98.8%)	373298 (98.7%)
A/WH/20230314-30/2023|A/H3N2	EPI_ISL_20239489	196676 (98.6%)	96742.9 (98.7%)	200951 (98.8%)	255930 (98.7%)	220299 (98.8%)	263692 (98.7%)	111692 (98.8%)	12238.1 (98.6%)
A/WH/20230314-33/2023|A/H3N2	EPI_ISL_20239497	49763.3 (97.7%)	44628.3 (96%)	98764.2 (98.3%)	4.25279 (66.3%)	108661 (98.7%)	125841 (98.7%)	315433 (98.6%)	280.823 (98.7%)
A/WH/20230321-12/2023|A/H3N2	EPI_ISL_20239512	211044 (98.7%)	128516 (98.7%)	208988 (98.8%)	235447 (98.7%)	41658.6 (98.6%)	74267.4 (98.7%)	400814 (98.7%)	139903 (98.6%)
A/WH/20230326-7/2023|A/H3N2	EPI_ISL_20239513	5029.72 (98.7%)	5094.65 (98.7%)	25423 (98.7%)	100771 (98.7%)	1580.62 (98.8%)	15937 (98.7%)	59496.1 (91.5%)	1797.05 (98.7%)
A/WH/20231122-1/2023|A/H3N2	EPI_ISL_20239514	128045 (98.7%)	441.944 (98.7%)	7681.86 (98.7%)	39811 (98.7%)	340.824 (98.7%)	0	22554 (98.7%)	9744.25 (46.9%)
A/WH/20231220-1/2023|A/H3N2	EPI_ISL_20239515	75117.2 (98.7%)	26405.9 (98.7%)	98615.7 (98.8%)	70090.3 (98.7%)	1615.65 (95.9%)	20288.5 (98.7%)	11867.3 (98.8%)	21909.1 (98.7%)

A/WH/20231220-2/2023|A/H3N2	EPI_ISL_20239774	46209.4 (98.7%)	70149.4 (98.7%)	13269 (98.8%)	15763 (98.7%)	23702 (98.8%)	4877.79 (98.7%)	119205 (98.6%)	411.595 (98.6%)
A/WH/20231220-3/2023|A/H3N2	EPI_ISL_20239775	25993.9 (98.7%)	27502.4 (98.8%)	20738 (98.8%)	37223.9 (98.7%)	0	71218.7 (98.7%)	158920 (91.6%)	31874.8 (98.7%)
A/WH/20231231-2/2023|A/H3N2	EPI_ISL_20239776	591904 (98.7%)	153173 (98.7%)	412092 (98.8%)	353817 (98.7%)	155441 (98.8%)	222617 (98.7%)	682591 (98.5%)	129800 (98.7%)
A/WH/20230308-14/2023|A/H3N2	EPI_ISL_20238020	170008 (98.78%)	101389 (98.8%)	200150 (98.80%)	459876(95.99%)	193804 (98.71%)	335859 (98.78%)	129126 (98.60%)	371016 (98.78%)

Supplementary Table S3. Amino acid substitutions in antigenic sites of HA gene of H1N1.

**Antigenic sites**		**Ca2**			**Sb**		**RBS**									
**Amino acid position**	**137**	**154**	**159**	**166**	**202**	**233**	**240**	**277**	**294**	**330**	**373**	**408**	**416**	**435**	**444**	**468**
**A/Victoria/4897/2022**	**T**	**S**	**R**	**I**	**I**	**A**	**R**	**E**	**A**	**R**	**D**	**T**	**H**	**V**	**V**	**H**
A/WH/20230309-5/2023||A/H1N1||2023-03-09||HA		P	K			T	Q	D	T		E					N
A/WH/20230310-13/2023||A/H1N1||2023-03-10||HA	A	P	K	V	V	T	Q	D	T		E			I		N
A/WH/20230310-2/2023||A/H1N1||2023-03-10||HA			K			T	Q	D	T		E					N
A/WH/20230311-6/2023||A/H1N1||2023-03-11||HA		P	K			T	Q	D	T		E	I			I	N
A/WH/20230314-27/2023||A/H1N1||2023-03-14||HA		P	K			T	Q	D	T		E					N
A/WH/20230314-9/2023||A/H1N1||2023-03-14||HA		P	K			T	Q	D	T	K	E		N			N
A/WH/20230319-6/2023||A/H1N1||2023-03-19||HA		T	K			T	Q	D	T		E					N
A/WH/20230320-9/2023||A/H1N1||2023-03-20||HA		P	K			T	Q	D	T		E					N
A/WH/20230326-4/2023||A/H1N1||2023-03-26||HA		P	K			T	Q	D	T		E					N

Supplementary Table S4. Amino acid substitutions in antigenic sites of HA gene of H3N2.

**Antigenic sites**			**M-N**			**E**								**A**	**A**	**A**		**A**	**A**		**B**	**B**	**B**	**B**
**Amino acid position**	**3**	**9**	**37**	**41**	**66**	**69**	**78**	**95**	**99**	**108**	**110**	**112**	**120**	**137**	**138**	**147**	**156**	**158**	**160**	**166**	**172**	**175**	**176**	**180**
**A/Hong Kong/4801/2014**	**T**	**Y**	**P**	**I**	**E**	**D**	**E**	**F**	**K**	**K**	**Y**	**S**	**D**	**N**	**N**	**T**	**I**	**R**	**S**	**R**	**H**	**Y**	**K**	**L**
A/WH/20230307-1/2023||A/H3N2||2023-03-07||HA	A	N	P		K	N	G	F	E	R	N			K	D	K	K	G			S	N	I	Q
A/WH/20230308-11/2023||A/H3N2||2023-03-08||HA		N	P			G	G	F	E	R	N	N	G	K	N	K		G			S	N	I	Q
A/WH/20230311-5/2023||A/H3N2||2023-03-11||HA	A	N	P		K	N	G	F	E	R	N			R		K	K	G			S	N	I	Q
A/WH/20230314-30/2023||A/H3N2||2023-03-14||HA	A	N	P		K	N	G	F	E	R	N			K	D	K	K	G			S	N	I	Q
A/WH/20230321-12/2023||A/H3N2||2023-03-21||HA	A	N	P		K	N	G	F	E	R	N			K		K	K	G			S	N	I	Q
A/WH/20230326-7/2023||A/H3N2||2023-03-26||HA		N	P			G	G	F	E	R	N	N	G	K		K	K	G			S	N	I	Q
A/WH/20231122-1/2023||A/H3N2||2023-11-22||HA	A	N	P		K	N	G	F	E	R	N			K		K	K	G			S	N	I	Q
A/WH/20231220-1/2023||A/H3N2||2023-12-20||HA	A	N	P	V	K	N	G	F	E	R	N			K		K	K	G			S	N	I	Q
A/WH/20231220-2/2023||A/H3N2||2023-12-20||HA	A	N	P		K	N	G	F	E	R	N			K		K	K	G	N		S	N	I	Q
A/WH/20231220-3/2023||A/H3N2||2023-12-20||HA	A	N	P		K	N	G	L	E	R	N			K	D	K	K	G			S	N	I	Q
A/WH/20231231-2/2023||A/H3N2||2023-12-31||HA	A	N	S		K	N	G	L	E	R	N			K	D	K	K	G		K	S	N	I	Q

**Antigenic sites**	**L. P**	**B**	**B**	**B**	**B**	**B**	**B**	**RBS**		**C**					**M-N**							
**Amino acid position**	**187**	**202**	**206**	**208**	**209**	**210**	**211**	**239**	**255**	**292**	**315**	**327**	**363**	**394**	**422**	**434**	**447**	**500**	**521**	**538**	**545**	**550**
**A/Hong Kong/4801/2014**	**N**	**G**	**D**	**I**	**F**	**P**	**Y**	**I**	**P**	**K**	**R**	**H**	**V**	**N**	**I**	**I**	**L**	**G**	**V**	**I**	**V**	**F**
A/WH/20230307-1/2023||A/H3N2||2023-03-07||HA	K	D	N	F	S	L	F	V				Q		S	V			E		M	I	
A/WH/20230308-11/2023||A/H3N2||2023-03-08||HA	K	D	N		S	L	F			R		Q			V			E		M	I	
A/WH/20230311-5/2023||A/H3N2||2023-03-11||HA	K	D	N	F	S	L	F	V				Q		S	V		M	E		M	I	
A/WH/20230314-30/2023||A/H3N2||2023-03-14||HA	K	D	N	F	S	L	F	V				Q		S	V			E		M	I	
A/WH/20230321-12/2023||A/H3N2||2023-03-21||HA	K	D	N	F	S	L	F	V				Q		S	V			E	I	M	I	
A/WH/20230326-7/2023||A/H3N2||2023-03-26||HA	K	D	N		S	L	F			R	K	Q			V			E		M	I	
A/WH/20231122-1/2023||A/H3N2||2023-11-22||HA	K	D	N	F	S	L	F	V				Q		S	V			E	I	M	I	
A/WH/20231220-1/2023||A/H3N2||2023-12-20||HA	K	D	N	F	S	L	F	V				Q	M	S	V	V		E		M	I	L
A/WH/20231220-2/2023||A/H3N2||2023-12-20||HA	K	D	N	F	S	L	F	V				Q	M	S	V			E	I	M	I	
A/WH/20231220-3/2023||A/H3N2||2023-12-20||HA	K	D	N	F	S	L	F	V	S	E		Q		S	V			E		M	I	
A/WH/20231231-2/2023||A/H3N2||2023-12-31||HA	K	D	N	F	S	L	F	V	S	E		Q		S	V			E		M	I	

continued

Supplementary Table S5. Amino acid substitutions in NA gene of influenza virus A(H1N1).

**Amino acid position**	**50**	**72**	**77**	**86**	**200**	**216**	**247**	**257**	**382**
**A/Victoria/4897/2022**	**D**	**T**	**R**	**A**	**S**	**I**	**S**	**R**	**E**
A/WH/20230309-5/2023‖A/H1N1‖2023-03-09‖NA	N	I	K	T	N				G
A/WH/20230310-13/2023‖A/H1N1‖2023-0310‖NA	N					V		K	G
A/WH/20230310-2/2023‖A/H1N1‖2023-03-10‖NA	N				N				G
A/WH/20230311-6/2023‖A/H1N1‖2023-03-11‖NA	N				N				G
A/WH/20230314-27/2023‖A/H1N1‖2023-0314‖NA	N		K	T	N				G
A/WH/20230314-36/2023‖A/H1N1‖2023-0314‖NA	N				N				G
A/WH/20230314-9/2023‖A/H1N1‖2023-03-14‖NA	N				N		N		G
A/WH/20230319-6/2023‖A/H1N1‖2023-03-19‖NA	N		K	T	N				G
A/WH/20230320-9/2023‖A/H1N1‖2023-03-20‖NA	N				N				G
A/WH/20230326-4/2023‖A/H1N1‖2023-03-26‖NA	N				N				G

Supplementary Table S6. Amino acid substitutions in NA gene of influenza virus A(H3N2).

Amino acid position	26	53	93	100	126	149	150	215	220	231	245	247	264	267	303	308	315	329	331	339	344	346	370	380	392	399	400	463	465	468	469
OQ350820.1/A/Hong Kong/4801/2014	I	C	G	F	P	V	R	V	K	I	S	S	H	T	V	K	S	N	S	D	E	G	S	I	T	D	R	D	N	P	I
A/WH/20230307-1/2023||A/H3N2||2023-03-07||NA					L		H		N	V	N	T	Y	K	I		R	S		N	K	D		V	I			N	S	H	T
A/WH/20230308-14/2023||A/H3N2||2023-03-08||NA					L		H		N	V	N	T		K	I		R	S		N	K	D		V	I			N	S	H	T
A/WH/20230311-5/2023||A/H3N2||2023-03-11||NA					L		H		N	V	N	T		K	I		R	S		N	K	D	A	V	I			N	S	H	T
A/WH/20230314-30/2023||A/H3N2||2023-03-14||NA					L		H	L	N	V	N	T		K	I		R	S		N	K	D		V	I			N	S	H	T
A/WH/20230314-33/2023||A/H3N2||2023-03-14||NA					L		H		N	V	N	T		K	I		R	S		N	K	D		V	I			N	S	H	T
A/WH/20230321-12/2023||A/H3N2||2023-03-21||NA					L	A	H		N	V	N	T		K	I		R	S	G	N	K	D		V	I			N	S	H	T
A/WH/20230326-7/2023||A/H3N2||2023-03-26||NA					L				N	V	N	T		K	I		R	G		N	K			V	I			N	S	H	
A/WH/20231220-1/2023||A/H3N2||2023-12-20||NA	T				L		H		N	V	N	T		K	I		R		G	N	K	D		V	M			N	S	H	T
A/WH/20231220-2/2023||A/H3N2||2023-12-20||NA			S	L	L		H		N	V	N	T		K	I		R	S	G	N	K	D		V	I			N	S	H	T
A/WH/20231220-3/2023||A/H3N2||2023-12-20||NA					L		H		N	V	N	T		K	I	R	R	S		N	K	D		V	I		K	N	S	H	T
A/WH/20231231-2/2023||A/H3N2||2023-12-31||NA		Y			L		H		N	V	N	T		K	I	R	R	S		N	K	D		V	I	G		N	S	H	T

Supplementary Table S7. N-glycosylation sites of HA and NA gene (A/H1N1).

	N-Glycosylation site	
**Gene**	**HA**	**NA**
**Position**	**293**	**50**
**A/Victoria/4897/2022**	**NAT**	**DKS**
A/WH/20230309-5/2023	**NTT**	**NKS**
A/WH/20230310-13/2023	**NTT**	**NKS**
A/WH/20230310-2/2023	**NTT**	**NKS**
A/WH/20230311-6/2023	**NTT**	**NKS**
A/WH/20230314-27/2023	**NTT**	**NKS**
A/WH/20230314-9/2023	**NTT**	**NKS**
A/WH/20230319-6/2023	**NTT**	**NKS**
A/WH/20230320-9/2023	**NTT**	**NKS**
A/WH/20230326-4/2023	**NTT**	**NKS**
A/WH/20230314-36/2023		**NKS**

Supplementary Table S8. N-glycosylation sites of HA and NA gene (A/H3N2).

	**N-Glycosylation site**						
**Gene**	**HA**	**HA**	**HA**	**HA**	**NA**	**NA**	**NA**
**Position**	**109**	**138**	**160**	**499**	**245**	**329**	**463**
**A/Hong Kong/4801/2014**	**YSS**	**NES**	**SSS**	**NGT**	**SAS**	**NDS**	**DLN**
A/WH/20230307-1/2023	YSS	**DES**	SSS	**NET**	**NAT**	**SDS**	**NLS**
A/WH/20230311-5/2023	**NSN**	NES	SSS	**NET**	**NAT**	**SDS**	**NLS**
A/WH/20230314-30/2023	YSS	**DES**	SSS	**NET**	**NAT**	**SDS**	**NLS**
A/WH/20230321-12/2023	YSS	NES	SSS	**NET**	**NAT**	**SDS**	**NLS**
A/WH/20230326-7/2023	**NSN**	NES	SSS	**NET**	**NAT**	**SDG**	**NLS**
A/WH/20231220-1/2023	YSS	NES	SSS	**NET**	**NAT**	**NDG**	**NLS**
A/WH/20231220-2/2023	YSS	NES	**NSS**	**NET**	**NAT**	**SDG**	**NLS**
A/WH/20231220-3/2023	YSS	**DES**	SSS	**NET**	**NAT**	**SDS**	**NLS**
A/WH/20231231-2/2023	YSS	**DES**	SSS	**NET**	**NAT**	**SDS**	**NLS**
A/WH/20230308-14/2023					**NAT**	**SDS**	**NLS**
A/WH/20230314-33/2023					**NAT**	**SDS**	**NLS**
A/WH/20231122-1/2023	YSS	NES	SSS	**NET**			
A/WH/20230308-11/2023	YSS	NES	SSS	**NET**			
