# Supplementary material for: Genomic Evolution of Influenza A(H1N1)pdm09 and A/H3N2 Viruses Among Children in Wuhan, China, Spanning the COVID-19 Pandemic (2020–2023)
Source: Viruses. 2026 Feb 5;18(2):210. doi: 10.3390/v18020210 (PMC12945282; doi:10.3390/v18020210)
Supplement: Supplementary file 1 [file viruses-18-00210-s001.zip › Table S9_Sample’s Isolate IDs & accession nos.pdf]

Supplementary Table S9. Sample's Isolate IDs & accession nos.

Sample's Isolate IDs & accession no. of subtype A/H3N2 uploaded on GISAID					
**Sr No.**	**Isolate ID**	**Sample Name**	**Segment**	**Accession No.**	**Subtype**
1	EPI_ISL_20238018	20230307-1	PB2	EPI4778176	H3N2
			PB1	EPI4778177	
			PA	EPI4778178	
			HA	EPI4778179	
			NP	EPI4778180	
			NA	EPI4778181	
			MP	EPI4778182	
			NS	EPI4778183	
2	EPI_ISL_20238019	20230308-11	PB2	EPI4778184	
			PB1	EPI4778185	
			PA	EPI4778186	
			HA	EPI4778187	
			NP	EPI4778188	
			MP	EPI4778189	
			NS	EPI4778190	
3	EPI_ISL_20238020	20230308-14	PB2	EPI4778191	
			PB1	EPI4778192	
			PA	EPI4778193	
			NP	EPI4778194	
			NA	EPI4778195	
			MP	EPI4778196	
			NS	EPI4778197	
4	EPI_ISL_20239487	20230311-5	PB2	EPI4782453	
			PB1	EPI4782454	
			PA	EPI4782455	
			HA	EPI4782456	
			NP	EPI4782457	
			NA	EPI4782458	
			MP	EPI4782459	
			NS	EPI4782460	
5	EPI_ISL_20239489	20230314-30	PB2	EPI4782461	
			PB1	EPI4782462	
			PA	EPI4782463	
			HA	EPI4782464	
			NP	EPI4782465	
			NA	EPI4782466	
			MP	EPI4782467	
			NS	EPI4782468	

6	EPI_ISL_20239497	20230314-33	PB2	EPI4782469
			PB1	EPI4782470
			PA	EPI4782471
			NP	EPI4782472
			NA	EPI4782473
			MP	EPI4782474
			NS	EPI4782475
7	EPI_ISL_20239512	20230321-12	PB2	EPI4782476
			PB1	EPI4782477
			PA	EPI4782478
			HA	EPI4782479
			NP	EPI4782480
			NA	EPI4782481
			MP	EPI4782482
			NS	EPI4782483
8	EPI_ISL_20239513	20230326-7	PB2	EPI4782484
			PB1	EPI4782485
			PA	EPI4782486
			HA	EPI4782487
			NP	EPI4782488
			NA	EPI4782489
			MP	EPI4782490
			NS	EPI4782491
9	EPI_ISL_20239514	20231122-1	PB2	EPI4782521
			PB1	EPI4782526
			PA	EPI4782530
			HA	EPI4782532
			NP	EPI4782535
			MP	EPI4782539
10	EPI_ISL_20239515	20231220-1	PB2	EPI4782567
			PB1	EPI4782575
			PA	EPI4782576
			HA	EPI4782582
			NP	EPI4782584
			NA	EPI4782588
			MP	EPI4782591
			NS	EPI4782594
11	EPI_ISL_20239774	20231220-2	PB2	EPI4782680
			PB1	EPI4782681
			PA	EPI4782682
			HA	EPI4782683
			NP	EPI4782684
			NA	EPI4782685

			MP	EPI4782686	
			NS	EPI4782687	
12	EPI_ISL_20239775	20231220-3	PB2	EPI4782688	
			PB1	EPI4782689	
			PA	EPI4782690	
			HA	EPI4782691	
			NA	EPI4782692	
			MP	EPI4782693	
			NS	EPI4782694	
13	EPI_ISL_20239776	20231231-2	PB2	EPI4782695	
			PB1	EPI4782696	
			PA	EPI4782697	
			HA	EPI4782698	
			NP	EPI4782699	
			NA	EPI4782700	
			MP	EPI4782701	
			NS	EPI4782702	

Sample's Isolate IDs & accession no. of subtype A/H1N1 uploaded on GISAID

Sr No.	Isolate ID	Sample Name	Segment	Accession No.	Subtype
1	EPI_ISL_20239865	20230309-5	PB2	EPI4783006	H1N1
			PB1	EPI4783007	
			PA	EPI4783008	
			HA	EPI4783009	
			NP	EPI4783010	
			NA	EPI4783011	
			MP	EPI4783012	
			NS	EPI4783013	
2	EPI_ISL_20239866	20230310-2	PB2	EPI4783014	
			PB1	EPI4783015	
			PA	EPI4783016	
			HA	EPI4783017	
			NP	EPI4783018	
			NA	EPI4783019	
			MP	EPI4783020	
			NS	EPI4783021	
3	EPI_ISL_20239867	20230310-13	PB2	EPI4783022	
			PB1	EPI4783023	
			PA	EPI4783024	
			HA	EPI4783025	
			NP	EPI4783026	

			NA	EPI4783027
			MP	EPI4783028
			NS	EPI4783029
4	EPI_ISL_20239868	20230311-6	PB2	EPI4783030
			PB1	EPI4783031
			PA	EPI4783032
			HA	EPI4783033
			NP	EPI4783034
			NA	EPI4783035
			MP	EPI4783036
			NS	EPI4783037
5	EPI_ISL_20239960	20230314-9	PB2	EPI4783766
			PA	EPI4783767
			HA	EPI4783768
			NP	EPI4783769
			NA	EPI4783770
			MP	EPI4783771
			NS	EPI4783772
6	EPI_ISL_20239961	20230314-27	PB2	EPI4783773
			PA	EPI4783774
			HA	EPI4783775
			NP	EPI4783776
			NA	EPI4783777
			MP	EPI4783778
			NS	EPI4783779
7	EPI_ISL_20239962	20230314-36	PB2	EPI4783780
			PB1	EPI4783781
			PA	EPI4783782
			NP	EPI4783783
			NA	EPI4783784
			MP	EPI4783785
			NS	EPI4783786
8	EPI_ISL_20239963	20230319-6	PB2	EPI4783787
			PB1	EPI4783788
			PA	EPI4783789
			HA	EPI4783790
			NP	EPI4783791
			NA	EPI4783792
			MP	EPI4783793
			NS	EPI4783794
9	EPI_ISL_20239964	20230320-9	PB2	EPI4783795
			PB1	EPI4783796
			PA	EPI4783797

			HA	EPI4783798
			NP	EPI4783799
			NA	EPI4783800
			MP	EPI4783801
			NS	EPI4783802
10	EPI_ISL_20239960	20230326-4	PB2	EPI4783803
			PB1	EPI4783804
			PA	EPI4783805
			HA	EPI4783806
			NP	EPI4783807
			NA	EPI4783808
			MP	EPI4783809
			NS	EPI4783810
